# Adaptive evolutionary trajectories in complexity: Transitions between unicellularity and facultative differentiated multicellularity

**DOI:** 10.1073/pnas.2411692122

**Published:** 2025-01-22

**Authors:** Hanna Isaksson, Peter Lind, Eric Libby

**Affiliations:** ^a^Department of Mathematics and Mathematical Statistics, Umeå University, Umeå 90187, Sweden; ^b^IceLab, Umeå University, Umeå 90187, Sweden; ^c^Department of Molecular Biology, Umeå University, Umeå 90187, Sweden; ^d^Umeå Centre for Microbial Research, Umeå University, Umeå 90187, Sweden

**Keywords:** multicellularity, differentiation, complexity, adaptation

## Abstract

Multicellularity spans a wide gamut in terms of complexity, from simple clonal clusters of cells to large-scale organisms composed of differentiated cells and tissues. While recent experiments have demonstrated that simple forms of multicellularity can readily evolve in response to different selective pressures, it is unknown if continued exposure to those same selective pressures will result in the evolution of increased multicellular complexity. We use mathematical models to consider the adaptive trajectories of unicellular organisms exposed to periodic bouts of abiotic stress, such as drought or antibiotics. Populations can improve survival in response to the stress by evolving multicellularity or cell differentiation—or both; however, these responses have associated costs when the stress is absent. We define a parameter space of fitness-relevant traits and identify where multicellularity, differentiation, or their combination is fittest. We then study the effects of adaptation by allowing populations to fix mutations that improve their fitness. We find that while the same mutation can be beneficial to populations of different complexity, e.g., strict unicellularity or life cycles with stages of differentiated multicellularity, the magnitudes of their effects can differ and alter which is fittest. As a result, we observe adaptive trajectories that gain and lose complexity. We also show that the order of mutations, historical contingency, can cause some transitions to be permanent in the absence of neutral evolution. Ultimately, we find that continued exposure to a selective driver for multicellularity can either lead to increasing complexity or a return to unicellularity.

## Significance

Our study explores how unicellular organisms evolve multicellularity and cell differentiation when subjected to abiotic stresses such as drought or antibiotics. Using mathematical models, we demonstrate that these complex forms can arise as adaptive strategies, enhancing survival and boosting population growth. However, their stability is not guaranteed, as populations often acquire beneficial mutations that render other traits unnecessary. Consequently, we observe both gains and losses in complexity, influenced by the order and nature of mutations. This research advances our understanding of how complex life can evolve and adapt in response to persistent environmental challenges.

There is a broad spectrum of multicellularity that ranges from primitive replicating groups of cells to large complex organisms with intricate tissues and behaviors (1–5). Empirical studies have demonstrated that unicellular organisms can readily evolve forms of multicellularity in response to many different selective pressures, including predation risk, drought, settling speed, salt stress, and many more (6–10). However, these forms of multicellularity are often relatively simple and lack the basic features of fully fledged multicellular organisms such as cell adhesion and cell–cell communication (11–13). Moreover, since these early forms of multicellularity are usually only one mutation away from unicellularity (14), they can easily revert should the selection pressure change. Even if the selection pressure for multicellularity is maintained, forms of complexity, such as cell differentiation, may not evolve or may fail to be incorporated in the multicellular stage. For example, choanoflagellates have a life cycle that differentiates between cell types and has multicellular stages but the multicellular stages are composed of only a single cell type (15, 16). Thus, despite the many selective drivers of multicellularity there appear to be obstacles that inhibit the evolution of further complexity, raising the question of whether selective drivers for multicellularity can also sustain the evolution of further complexity or if there are fundamental challenges along the way that limit the evolution of complexity.

Given the breadth of multicellular forms and types of complexity, we narrow the scope of the question by focusing on the evolutionary path from a strict unicellular life cycle to a life cycle that includes multicellular stages that incorporate differentiation, referred to here as “differentiated multicellularity.” In these early stages of multicellular complexity, populations are genetically close to their unicellular ancestors and differentiation and multicellularity often arise as transient responses to environmental triggers. These stages differ from more established multicellularity seen in animals and plants, where multicellularity is a dominant and obligate part of the life cycle and differentiation is mediated by cell–cell signaling and developmental programs. By concentrating on the early, precarious steps, we can examine how selective pressures filter evolutionary paths, potentially guiding or limiting which forms of multicellularity and differentiation persist, influencing the emergence of later, more stable forms of complexity.

Since the evolution of differentiated multicellularity involves two innovations, there are different possible combinations of selective regimes. One possible regime, often called “differentiation first,” draws on the observation that many unicellular organisms have the ability to express different phenotypes, or cell types, in order to switch between tasks in time (17, 18). During the subsequent evolution of multicellularity the different cell types could then be arranged in spatial patterns, co-opted for new functions that support the nascent multicellular organism (19–21). Another possibility is that multicellularity evolved first and then generated a new context that allowed the evolution of de novo cell types. For some model systems, this involves modification of a trait already present in unicellular populations but not necessarily exploited, e.g., increasing cell death rate (22, 23); while in other model systems differentiation evolves by partitioning two tasks typically performed simultaneously in unicellular organisms across two different cells (24, 25). Theoretical studies have explored the “multicellularity first” route by identifying conditions that allow for differentiation to evolve within a multicellular context (26–29). In these models differentiation usually manifests as a division of labor where reproductive or metabolic/energetic tasks are split between cells. Based on these models a selective regime necessary to produce differentiated multicellularity is clear: selection for multicellularity can also lead to differentiated cell types that cooperate to increase multicellular fitness.

A problem with these approaches is that they typically consider the evolution of differentiated multicellularity within a limited parameter space for adaptation. For instance, by studying whether differentiation can evolve within a multicellular context, populations are constrained so that adaptation occurs only along the axis of differentiation, i.e., evolving it or not. Yet, adaptation can occur across many more dimensions in the same environment that selects for multicellularity or differentiation. Although some of these adaptations may support multicellularity or differentiation, they can also reduce their benefits to the point that they are no longer advantageous—and even selected against. For example, in response to antibiotics bacterial populations can improve their survival by differentiating into special survival phenotypes or by forming multicellular biofilms (30–32). They can also improve their survival by evolving resistance to the antibiotic, in which case the plastic response to the antibiotic becomes maladaptive given the growth disadvantage of differentiation or biofilm formation (33). Thus, exposure to the same selective pressure that drives the evolution of differentiation and/or multicellularity could later lead populations to evolve resistance and revert back to their simpler, unicellular forms.

The example of antibiotics as a driver for both the evolution of complexity and its loss may be more generally applicable. We can more broadly categorize antibiotics as a type of abiotic stress where “abiotic” signifies that the stress does not evolve in response to the affected population(s). Other examples of abiotic stress include desiccation (34–36), high and low pH (37–39), salt (16, 40), etc., all of which threaten the survival of unicellular organisms but do not coevolve with them. We draw a distinction with biotic stress, e.g., predation, in which coevolution of two species may lead to changes in responses over time, e.g., the well-known arms races (41, 42). Abiotic stress has been identified as a potent selector for the evolution of simple multicellularity (43). In addition to multicellularity, abiotic stress may also select for types of differentiation. Bacterial persistence and bet hedging more generally have been shown to be favored in environments with regular bouts of abiotic stress (44–49). Although both multicellularity and differentiation can improve survival under abiotic stress, unicellular organisms may evolve other responses that mitigate the severity of the stress. Such responses include reduction or repair of molecular damage (e.g., oxidative stress responses (50, 51), DNA repair systems (52) and chaperones (53, 54) or modification of cellular constituents for example by alteration of membrane composition (55, 56) or accumulation of compatible solutes like trehalose (57).

Here we study the evolutionary trajectory from unicellularity toward an early multicellular life cycle with differentiation in a selective environment where populations experience repeated bouts of abiotic stress. In response to the stress cells may improve survival by forming multicellular groups or differentiating into survival specialists, or both. We use a mathematical modeling framework to weigh the benefits of survival from life cycles implementing these types of complexity versus the costs they impose to growth, manifesting as time delays as cells switch between types or forms. We explore the evolutionary trajectories when populations can adapt in multiple ways including increasing their growth rates or reducing the costs associated with differentiation or multicellularity. From these evolutionary trajectories, we analyze the stability of the evolved forms of complexity and determine whether adaptation leads to increasing multicellular complexity or reversion to unicellularity. Through our analyses, we find that both multicellularity and cell differentiation can evolve under the same abiotic stress, but not necessarily in the same life cycle. We also find that the path taken and its stability depend heavily on historical contingency and stochasticity, with the possibility of adaptive gains and losses of complexity. Thus we conclude that abiotic stress may well drive the evolution of differentiated multicellularity, but it may only appear in transient episodes along a path that ultimately returns to unicellularity.

## Results

### Model Description.

**Fig. 1.**
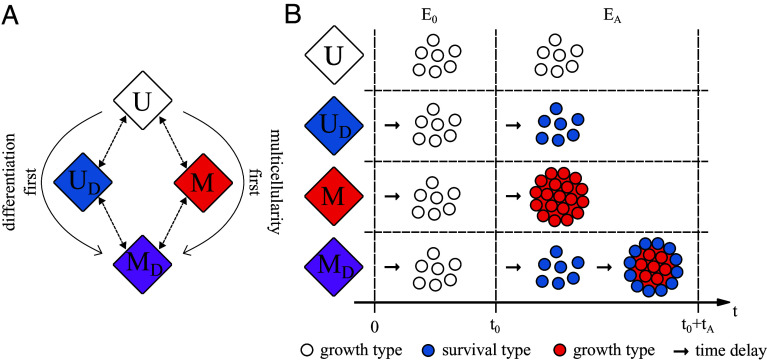
Evolutionary paths to a life cycle with an early form of differentiated multicellularity. (*A*) A schematic shows two different evolutionary routes from unicellularity (U) to differentiated multicellularity ($M_{D}$) depending on whether unicellular populations evolve differentiation or multicellularity first. (*B*) A schematic shows how different populations respond to fluctuations between environments without stress ($E_{0}$) and environments with an abiotic stress ($E_{A}$). The strictly unicellular population (U, white cells) grows during $E_{0}$ but then dies during exposure to the stress $E_{A}$. By differentiating into a survival phenotype ($U_{D}$, blue cells) cells become resistant to the stress. Another way to increase survival in response to the stress is to form multicellular groups (M, red cells), where the outer cells provide physical protection for inner cells. Differentiation and group formation can also be combined in a joint lifestyle, where cells form groups and the outer cells differentiate ($M_{D}$, blue and red cells). Each stress response comes with a cost in the form of time delays (represented by arrows).

Our aim is to explore how selection acting through an abiotic stress shapes the evolutionary paths leading from strict unicellularity to a life cycle with cell differentiation in its multicellular stages. In particular, we are interested in whether differentiation and group formation can both evolve, and how adaptation affects the evolutionary trajectories and their stability; see Fig. 1*A*. We consider a model in which an initial unicellular population experiences an environment that fluctuates between two states: 1) a neutral state ($E_{0}$) where the population grows exponentially according to a rate b and 2) a state containing an abiotic stress ($E_{A}$) that causes the population to die at a rate d. For simplicity, we assume that the time spent in each environment is the same, similar to the approach in ref. 58, but we explore the sensitivity of our results to this assumption in *SI Appendix*, *Sensitivity Analysis for the Environmental Duration*. In this model, the fitness of a population depends on the product of growth in $E_{0}$ and survival in $E_{A}$.

**Fig. 2.**
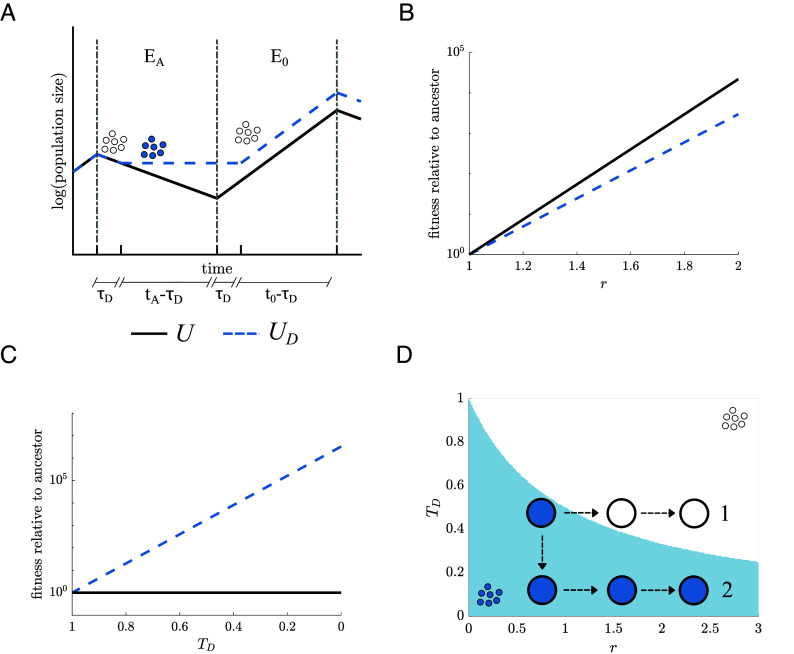
Fitness advantage of differentiation and evolutionary trajectories. (*A*) A schematic illustrates the dynamics of two populations in an environment that switches between two states: with and without an abiotic stress, $E_{A}$ and $E_{0}$, respectively. The first population is unicellular (U, solid line) which grows exponentially in $E_{0}$ and decays in $E_{A}$. The second population ($U_{D}$, blue dashed line) is similar to the first except that it differentiates to a stress-resistant phenotype that survives the stress in $E_{A}$ but can not reproduce. Differentiated populations experience a time lag $\tau_{D}$ each time they switch to and from the stress-resistant phenotype. (*B*) The two lines show how beneficial mutations in the birth–death ratio (r) change the fitness relative to an ancestor (r=1) in U (solid line) and $U_{D}$ (blue dashed line) populations. Beneficial mutations in r always cause larger fitness gain in U than $U_{D}$. (*C*) A plot shows the effects of beneficial mutations in the normalized time delay $T_{D}=\tau_{D}/t_{A}$. Decreasing $T_{D}$ increases the fitness of $U_{D}$ relative to its ancestor ($T_{D}=1$) but has no effect on U. For the calculations in this study, we have set the time spent in each environment ($t_{0}$ and $t_{A}$ for $E_{0}$ and $E_{A}$ respectively) to 10 time units. (*D*) A parameter space shows the values of the traits r and $T_{D}$ and is colored according to which is fitter U (white) or $U_{D}$ (blue). Populations undergo adaptive walks in parameter space via beneficial mutations in $T_{D}$ and r. Two example trajectories show that the type and order of mutations determine whether a $U_{D}$ population will maintain differentiation (trajectory 2) or cross into a region of the parameter space where loss of differentiation is favored and mutations in $T_{D}$ are neutral (trajectory 1).

Populations can improve their fitness by evolving strategies that better protect them from the abiotic stress; see Fig. 1*B*. One way for cells to do this is through differentiation into a specialized survival phenotype in the stressful environment. We assume that the survival phenotype allows cells to completely survive the stress but in this differentiated state they cannot reproduce—analogous to the flagellation constraint in Volvocales where cell division and flagellated motility can not take place simultaneously (59). We also assume that differentiating between phenotypes comes with a cost to cell growth. The cost manifests as a time lag $\tau_{D}$ when cells switch phenotypes; so when the environment switches states from stressful to neutral, there is a mismatch between the phenotype and the environment leading to a temporary reduction in growth (60, 61); see Fig. 2*A*. Thus, the fitness of this life cycle which we call “differentiated unicellularity” is determined by the interplay between two factors: the birth–death ratio and the time delay from switching phenotypes.

Another way for cells to improve their fitness during stress ($E_{A}$) is by forming multicellular groups, which provide physical protection to some interior set of cells (43, 62, 63). We choose to focus on multicellularity formed via aggregation, since this group-forming strategy responds faster to environmental changes than other types of multicellularity, e.g., clonal multicellularity where cells remain in groups (64). We assume that in the aggregated group, cells are partitioned into exterior and interior compartments such that cells on the exterior directly experience the abiotic stress while those in the interior are temporarily shielded from the stress (Fig. 1*B*). In the stressful environment, neither external nor internal cells can reproduce, which is common in aggregative multicellularity (65). For simplicity, we also assume that upon exposure to the stress exterior cells die at the same rate as if they were unicellular and then are immediately replaced by interior cells until there are no interior cells left. Thus, forming multicellular groups does not offer permanent protection but it does reduce the population death rate. Finally, we assume that multicellularity has a cost, similar to differentiation, which appears as a time lag $\tau_{G}$ between forming and breaking apart groups; so as in the case of differentiation, there is a trade-off between increased survival in $E_{A}$ and missed growth opportunities in $E_{0}$ (for details, see *Equations for Population Growth*).

A final strategy for improving fitness during abiotic stress combines cell differentiation with multicellularity, to produce a nascent form of differentiated multicellularity ($M_{D}$). While in principle there are many ways to combine differentiation with multicellularity (see *SI Appendix*, *Derivation of Differentiated Multicellularity* for other alternatives), we consider a way that minimizes interference and gives rise to complementary benefits. We assume that upon exposure to the abiotic stress cells first differentiate and then form groups. Since cells in the interior of the group are protected from the stress, they do not die and require replacement. This allows interior cells to dedifferentiate so that when the stress disappears they can rapidly start growing once the multicellular group is disbanded—they do not experience the time lag for differentiation in the growth environment. When leaving the stressful environment, groups first dissociate. Afterward, inner cells can start growing immediately, while outer cells must first dedifferentiate into the growth type. Thus, this life cycle which we call differentiated multicellularity has the potential to harness the benefits of both differentiation and group formation, but it also incurs the combined costs.

With our modeling framework, we can directly compute the fitness of populations as the net change across the two environments $E_{0}$ and $E_{A}$. Additional details of the implementation of our modeling framework can be found in *Equations for Population Growth*. In the subsequent sections, we consider what conditions favor the evolution of cell differentiation, multicellularity, or both.

### Differentiated Unicellularity.

Using a simple modeling framework of exponentially growing populations (*Equations for Population Growth*), we can identify the conditions for which differentiation confers a fitness advantage, i.e., differentiated unicellularity (denoted $U_{D}$) is fitter than undifferentiated unicellularity (denoted U). We let r represent the birth–death ratio and $T_{D}$ represent the time lag for differentiation ($\tau_{D}$) scaled by the time spent in $E_{A}$—we note that $T_{D}$ also represents the fraction of time in $E_{A}$ when cells are not protected. We solve for the expected growth of both U and $U_{D}$ populations (Eqs. **5** and **6**, respectively, in *Equations for Population Growth*) and find that differentiation provides a fitness advantage when

[1] $$\begin{array}{c} T_{D}<\frac{1}{1+r}. \end{array}$$

Eq. **1** shows that the value of r limits which time lags allow differentiation to be fitter. Since r is a ratio of the population growth rate in $E_{0}$ and the death rate in $E_{A}$, it encapsulates both the loss in growth and the gain in survival from switching. If r is low, either because populations grow slowly without stress or because the abiotic stress is particularly challenging, differentiation with high time lags can be advantageous.

One consequence of Eq. **1** is that adaptation can alter whether differentiation is favored. If we consider adaptation in terms of mutations that increase the value of r, either by improving the growth rate in $E_{0}$ or survival in response to the abiotic stress, fitness increases in both U and $U_{D}$; see Fig. 2*B*. However, in relative terms, r mutations increase fitness more in U so that continually increasing r will eventually cause unicellularity to become fitter than differentiated unicellularity. In contrast, if we consider adaptation in terms of mutations that lower the time delay in switching this only has a fitness effect on $U_{D}$ life cycles; see Fig. 2*C*. Lowering the time delay allows differentiation to be favored even for large values of r.

A result of these findings is that although differentiated unicellularity can improve fitness by either mutations in r or $T_{D}$, the order of these mutations can alter whether differentiation is maintained or lost. Fig. 2*D* shows that a mutation that lowers the time delay (resulting in lower $T_{D}$) followed by one that increases the birth–death ratio r can keep an organism with differentiation in areas of parameter space (r, $T_{D}$) where differentiation is fittest. Yet, if a mutation occurs in r first it can lead the population into an area of parameter space where unicellularity is fittest. Reversion to a strictly unicellular life cycle would then mean that any further mutations in $T_{D}$ are neutral and may not fix, preventing the population from evolving to an area of parameter space where differentiation is fitter.

### Multicellularity.

**Fig. 3.**
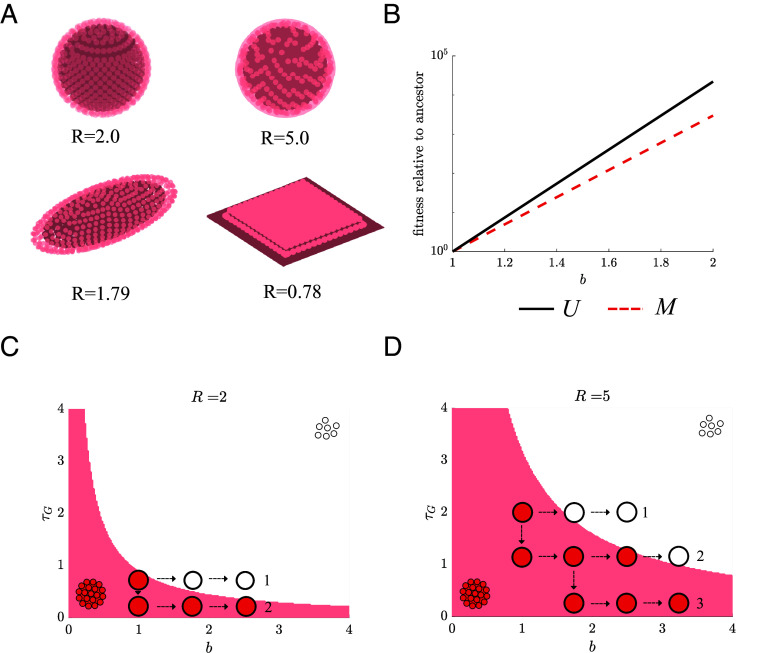
The dependence of the evolution of multicellularity on group topology and the order of mutations. (*A*) A schematic shows various multicellular group shapes, consisting of an outer cell layer (red cells) and a pool of inner cells (gray cells). The ratio of inner to outer cells, R, determines the group’s ability to protect cells from stress, where higher values of R indicate more protection. We show four example shapes and their corresponding R ratios: a tightly packed sphere (R=2.0), a sphere with its surface half-filled by cells (R=5.0), a rod-shaped group (R=1.79), and a 2D sheet similar to a biofilm (R=0.78). (*B*) A plot shows how beneficial mutations in the birth rate b have a larger positive impact on the fitness of populations of U (black solid line) compared to M (red dashed line). In the figure, relative fitness is compared to an ancestor with b=1. (*C*) A plot of the boundary from Eq. **2** that divides the parameter space into two regions, where either U (white) or M (red) has higher fitness. Beneficial mutations in $\tau_{G}$ and b enable populations to move in this space and possibly cross the boundary between fitness regions. The type and order of mutations influence whether a population will maintain multicellularity (trajectory 2) or revert to unicellularity (trajectory 1). (*D*) A plot shows the same boundary as in (*C*), but for a higher R value. Increasing R provides an advantage to multicellular groups and expands the region where M is favored and can be maintained.

Similar to the case of differentiation, we can derive a criterion for when a life cycle with a multicellular stage (M) has a fitness advantage over a strictly unicellular life cycle (U). The fitness advantage of M depends on how much protection is afforded to inner cells, which in turn depends on the structure of the group as well as the length of time spent in the stressful ($E_{A}$) environment. We characterize different group structures by introducing a parameter R which is the ratio of the number of interior cells to exterior cells when the group is first formed; higher values of R indicate structures with a higher fraction of protected interior cells. Since we do not explicitly impose a type of topology, the physical structure of the group can vary across R values; see Fig. 3*A*. To simplify analyses, we assume that the stress persists for enough time that the groups no longer contain inner cells (see *SI Appendix*, *Derivation of Undifferentiated Multicellularity* for analyses of different stress durations). Based on this assumption, we compute the expected growth of both populations (see Eq. **5** for U and $M_{2}$ in Eq. **7** for M in *Equations for Population Growth*) and find that M has higher fitness than U when

[2] $$\begin{array}{c} b\tau_{G}<R-\ln\left(1+R\right). \end{array}$$

Here, $b\tau_{G}$ expresses the cost of being multicellular in terms of the missed growth potential when groups break apart. The cost of being multicellular must be offset by the protection afforded, which is represented by the R−ln(1+R) term on the right hand side of Eq. **2**. By reducing the time lag, populations can lower the cost of being multicellular; however, if populations increase the birth rate b then this favors unicellularity more than multicellularity; see Fig. 3*B*.

By constructing a parameter space for b and $\tau_{G}$ and calculating the relative fitness for M vs. U we can again see how the order of beneficial mutations affects evolutionary trajectories. When group protection (R) is low early mutations in the birth rate b can lead populations into the region where U is fittest. Since mutations in the time delay for group formation ($\tau_{G}$) are neutral to U and may not fix, populations can get trapped in the unicellular life cycle, similar to the case with differentiation. If instead, early mutations occur in $\tau_{G}$, it is possible to lock populations into a region where multicellularity is fittest; see Fig. 3*C*. These trajectories are also shaped by the structure of the group, as higher values of R produce larger areas of parameter space (in terms of b and $\tau_{G}$) where M is favored; see Fig. 3*D*.

### Differentiated Multicellularity.

Now we consider when the combination of differentiation and multicellularity ($M_{D}$) confers a fitness advantage compared to either in isolation. We compute the expected growth of an $M_{D}$ population (Eq. **8** from *Equations for Population Growth*) and derive when it is fitter than a population with just differentiation (Eq. **3**) or just multicellularity (Eq. **4**).

[3] $$\begin{array}{c} \frac{R}{e^{b\tau_{G}}\left(R+1\right)-1}>e^{-b\tau_{D}}, \end{array}$$

[4] $$\begin{array}{c} e^{R-d(t_{A}-\tau_{D})}-R<e^{-b\tau_{D}}. \end{array}$$

Since the resulting criteria contain more than two parameters, visualizations of the adaptive trajectories depend on the choice of parameters. In *SI Appendix*, *Comparing Fitness Conditions for $U_{D}$ vs. $M_{D}$* we consider all pairwise choices for parameters in Eq. **3** for $M_{D}$ vs. $U_{D}$. These pairwise plots show that adaptation in some traits such as the growth rate b can have little effect on which strategy is fitter. Adaptation in other traits such as the differentiation time delay $\tau_{D}$ can cause all initial conditions favoring $M_{D}$ to eventually enter a region where $U_{D}$ is fitter.

In the case of differentiated multicellularity vs. multicellularity the criterion determining which is fittest also depends on several parameters (*SI Appendix*, *Comparing Fitness Conditions for M vs. $M_{D}$*). One difference with this criterion is that it draws a distinction between the growth rate b and the death rate d. We find that adaptation in b can lead to increased relative fitness of $M_{D}$ while adaptation in d can have the opposite effect, eventually leading to an area of parameter space where M is fitter. So adaptation in the birth–death ratio can favor either $M_{D}$ or M depending on which term is affected.

**Fig. 4.**
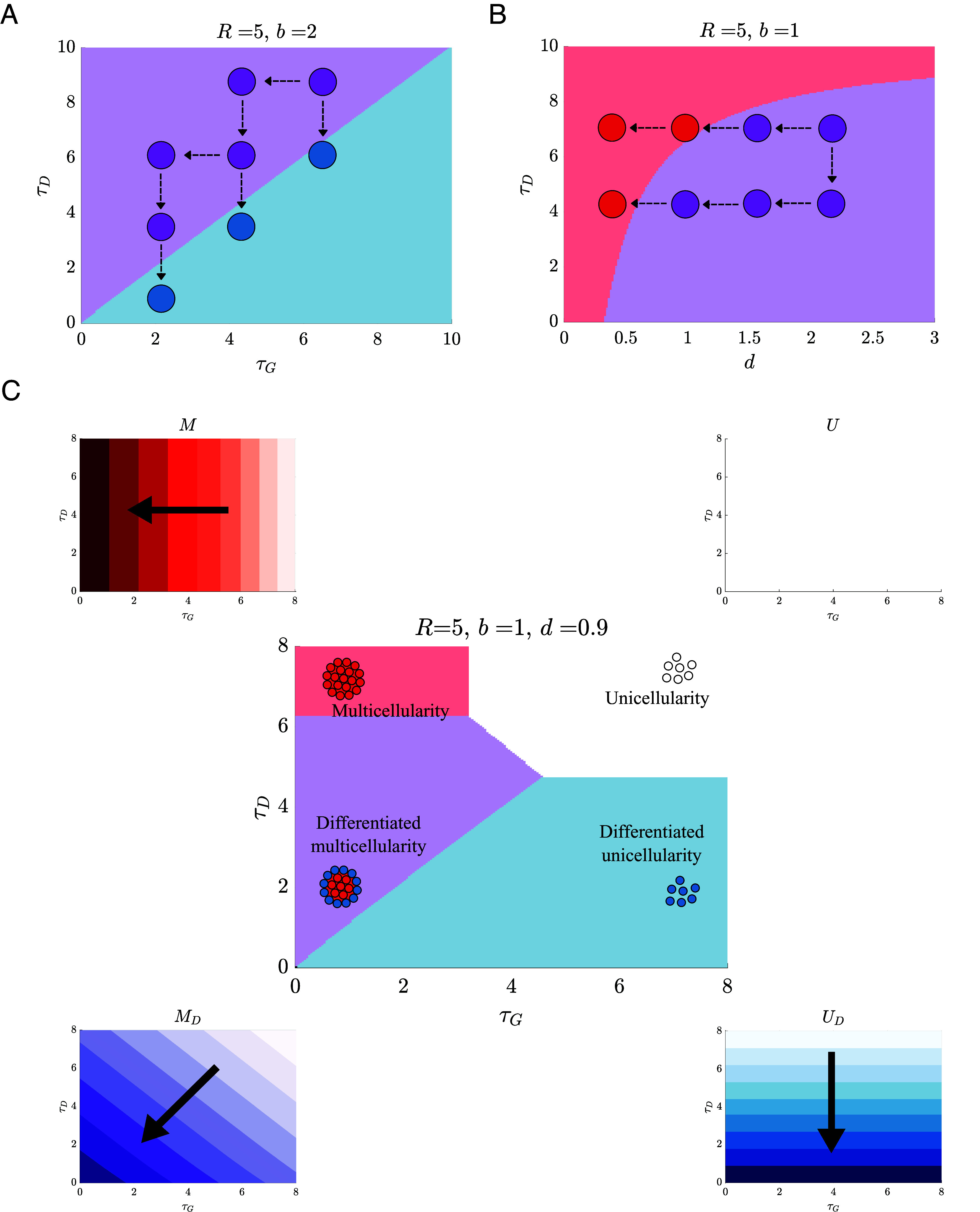
The many adaptive paths to and from a life cycle with differentiated multicellularity. (*A*) A plot of the boundary from Eq. **3** in the $\tau_{G}$–$\tau_{D}$ plane. While beneficial mutations that decrease $\tau_{G}$ lead to entrenchment of the $M_{D}$ life cycle (purple), mutations that decrease $\tau_{D}$ eventually lead to reversion to $U_{D}$ (blue). (*B*) A plot of the boundary from Eq. **4** in the d–$\tau_{D}$ plane. Beneficial mutations that reduce d eventually cause populations expressing $M_{D}$ (purple) to revert to M (red), losing differentiation. In contrast to (*A*) beneficial mutations that decrease $\tau_{D}$ reinforce $M_{D}$ and prevent reversion to M. (*C*) In the *Middle* panel, a plot shows the $\tau_{G}$–$\tau_{D}$ plane divided into regions where each life cycle has the highest fitness. The smaller surrounding panels illustrate how the fitness of each life cycle depends on $\tau_{G}$ and $\tau_{D}$ for example, U maintains the same fitness throughout the parameter space, while the fitness of $M_{D}$ increases as either time lag is shortened.

The conditions for when differentiated multicellularity is fitter than its evolutionary antecedents (Eqs. **3** and **4**) share some trait parameters, such as the time delay for differentiation $\tau_{D}$. If we consider beneficial mutations that decrease $\tau_{D}$ we find different effects on the relative fitness of differentiated multicellularity compared to differentiation $U_{D}$ and multicellularity M alone. Fig. 4*A* shows that decreasing the delay $\tau_{D}$ causes $U_{D}$ to be fitter than $M_{D}$, favoring a loss of multicellularity. In contrast, Fig. 4*B* shows that when d is not small (i.e., d>0.35) decreasing the delay $\tau_{D}$ causes $M_{D}$ to be fitter than M, favoring differentiation. These different results concerning the stability of $M_{D}$ in response to adaptation in the same trait occur because decreasing the time delay for differentiation alters the relative benefits of differentiation vs. multicellularity, which in turn alters the relative fitness of $M_{D}$ compared to $U_{D}$ and M.

We can combine the fitness calculations of the two types of complexities and the various life cycles (U, $U_{D}$, M, and $M_{D}$) to identify which is fittest for a given combination of parameters. Fig. 4*C* shows the results of this calculation for parameters $\tau_{G}$ and $\tau_{D}$ as well as the direction in parameter space where each life cycle increases fitness. Although each life cycle has a region where it is fittest, the regions for multicellularity and differentiated multicellularity are smaller than the region for differentiated unicellularity. If only beneficial mutations are considered then all life cycles except for $M_{D}$ are evolutionary stable in this parameter space. Differentiated multicellularity may remain the fittest provided the time delay for group formation is greater than differentiation; otherwise, it will cross into a region where $U_{D}$ is fittest. We note that in Fig. 4*C* we set R=5 to characterize the protection offered by multicellularity, but changing this value can alter the relative areas where each life cycle is fittest. In *SI Appendix*, *Sensitivity Analysis for the Group Parameter R* we show that reducing R to R=0.5 removes the region where multicellularity alone is favored while increasing R to R=10 reduces the region where $M_{D}$ is favored.

### Evolutionary Simulations.

**Fig. 5.**
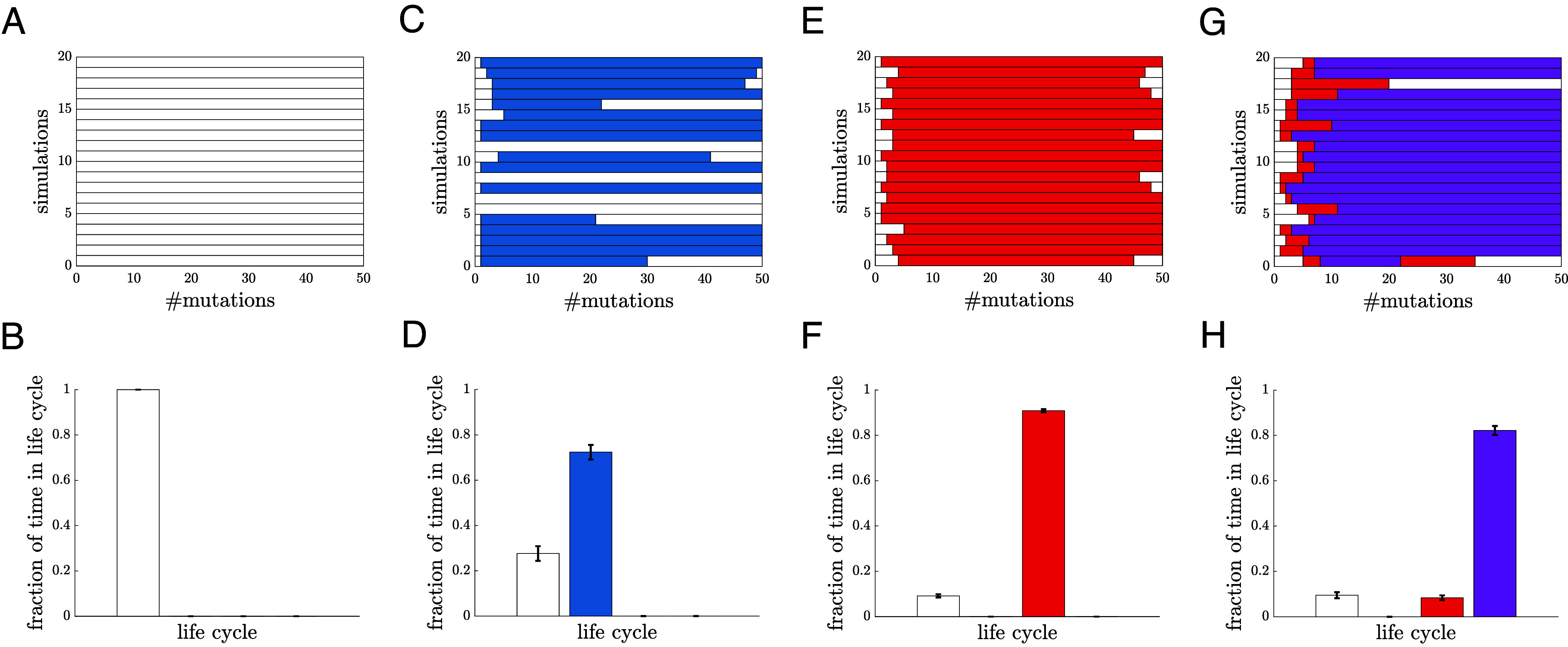
Historical contingency and the evolution of complexity. (*A*) A plot shows 20 sample evolutionary simulations. All mutations are adaptive and a mutation is accepted with a probability based on its relative fitness Eq. **9**. The initial parameters for panels (*A*–*D*) are listed in *SI Appendix*, *Sensitivity to Initial Conditions*. For the initial parameter setting used in (*A*), only the U life cycle (white) is observed. (*B*) A histogram shows the fraction of time spent in each life cycle; here, all time is spent as U. (*C*) A plot shows the evolutionary outcomes for a different initial parameter setting, where some simulations result in the evolution of $U_{D}$ (blue). (*D*) A histogram shows that more time is spent in the $U_{D}$ life cycle than U. (*E* and *F*) Panels show similar data as (*C* and *D*), but for an initial condition, that frequently results in the evolution of M (red). (*G*) A plot shows the data from simulations with an initial setting where M (red) and $M_{D}$ (purple) evolve. Since it takes two mutations, one in the ability to form groups and one to differentiate, we never observe a transition directly from U to $M_{D}$. (*H*) A histogram shows that $M_{D}$ is the most frequently observed life cycle given the initial parameter setting for (*G*).

Until now we have considered adaptive evolutionary trajectories in regard to two traits at a time, but Eqs. **3** and **4** show that the relative fitness of differentiated multicellularity $M_{D}$ compared to multicellularity M or differentiation $U_{D}$ depends on more parameters. To map possible adaptive trajectories in this higher dimensional parameter space, we perform evolutionary simulations that allow mutations in the defining traits of the life cycles (*Evolutionary Simulations*). More specifically, we consider mutations in the birth and death rates, the time delay for differentiation, and the time delay for group formation/dissociation. We also allow mutations to alter whether populations differentiate or form multicellular groups. For each mutation, we compute its fitness effect on the population and assume it fixes based on the extent to which it increases relative fitness (*Evolutionary Simulations*). All simulations start with a unicellular life cycle and we simulate 10,000 independent evolutionary trajectories for different sets of initial parameters. Fig. 5 shows that for different sets of initial parameters we can bias evolution toward different types of complexity. For example, by lowering the initial cost of differentiation, we can increase the frequency of evolutionary trajectories that evolve differentiation, U to $U_{D}$. *SI Appendix*, *Sensitivity to Initial Conditions* contains analyses for a more extensive set of initial parameters, which demonstrate that small changes in the initial values can alter the observed adaptive trajectories.

**Fig. 6.**
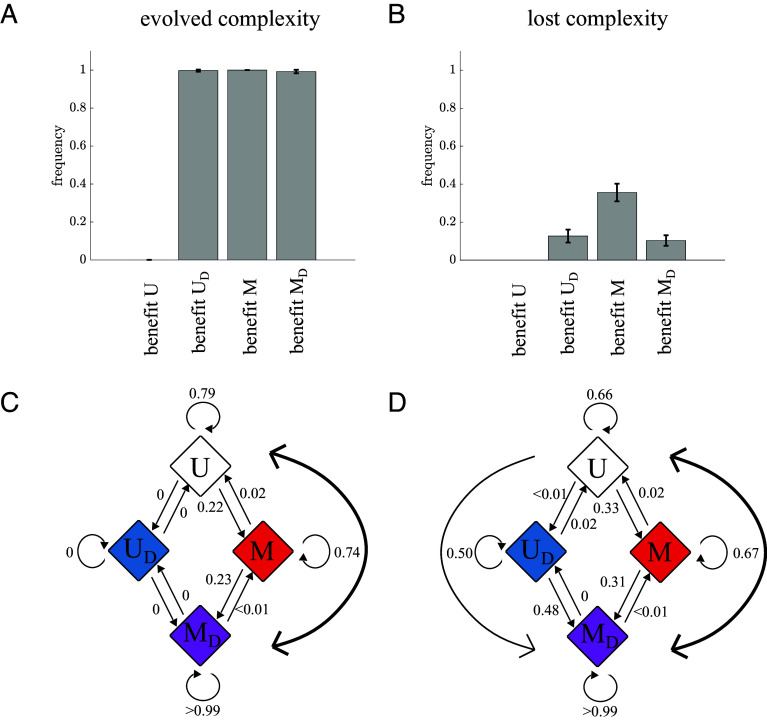
Gain and loss of complexity under constant selection pressure. (*A*) A histogram shows the fraction of simulations where either differentiation or multicellularity evolved starting from each of the initial parameter settings used in Fig. 5. In over 99% of all simulations—except those using the first set of parameters—some form of complexity evolved. (*B*) A histogram shows the fraction of simulations that reverted back to U, after first evolving some form of complexity. Between 10 to 35% of simulations lost complexity and reverted back to a strictly unicellular life cycle. (*C*) A diagram represents the transition probabilities between life cycles for the simulations in Fig. 5*G*. The data show that the most frequent path from U to $M_{D}$ evolves multicellularity first. Similarly reversions from $M_{D}$ to U happen by populations first losing the ability to differentiate. (*D*) A diagram represents transition probabilities for the same case as in (*C*), but neutral mutations are accepted with 20% probability. Neutral mutations alter the evolutionary dynamics by increasing the evolution of $M_{D}$ via multicellularity first (M) and differentiation first ($U_{D}$).

The trajectories in Fig. 5 also indicate that there can be adaptive gains and losses of complex traits. By choosing different sets of initial parameters we can bias adaptation to evolve some form of complexity ($U_{D}$, M, or $M_{D}$) in over 99% of simulations (Fig. 6*A*). Yet in 10 to 35% of the trajectories that evolved complexity, they ultimately reverted to a strictly unicellular life cycle by the end of the simulation; see Fig. 6*B*. These losses of complexity occurred because although all mutations increased fitness, mutations in some traits, such as the death rate d, increased the fitness of unicellularity more.

We can use the data from the simulated evolutionary trajectories to determine the characteristic paths for gaining and losing complexity. Here, we focus on the parameter setting that favored the evolution of differentiated multicellularity. In Fig. 6*C* we display the evolutionary paths between U, $U_{D}$, M, and $M_{D}$ as a state diagram where the arrows between states indicate transitions from one life cycle to another. This representation shows that when the differentiated multicellularity life cycle evolves it typically proceeds from U to M to $M_{D}$, i.e., it more often evolves multicellularity first rather than differentiation. Similarly, when differentiated multicellularity reverts back to a strictly unicellular life cycle, it typically follows the same path in reverse, from $M_{D}$ to M to U. We can alter the observed evolutionary paths from unicellularity to differentiated multicellularity by allowing neutral mutations to fix. Fig. 6*D* shows that when we permit neutral mutations to fix at a high rate, e.g., 20%, the trends from Fig. 6*C* are weakened as populations are freer to explore alternative paths to and from complexity (see *SI Appendix*, *Neutral Mutations* for the effects of neutral evolution on other initial parameter settings).

The state diagrams pertain only to evolutionary simulations using a specific set of initial parameters. In *SI Appendix*, *Alternative Evolutionary Trajectories* we show state diagram representations for other initial parameter settings in which a majority of simulations evolved differentiated multicellularity. We find that for these settings the characteristic paths from U to $M_{D}$ are different, going via $U_{D}$ or both $U_{D}$ and M. Thus historical contingency, represented here by the initial parameter values, determines the characteristic paths toward complexity.

## Discussion

Multicellularity has evolved independently in different lineages (3, 5, 13, 66, 67), and in many cases, it has come with, or led to, further gains in complexity such as specialized cell types (12, 59, 65, 68, 69) or advanced body architectures (70–72). There are also examples of multicellularity that have remained relatively simple or were lost as populations reverted back to their ancestral unicellular state. Investigations into the origins of complex multicellularity have typically considered features in isolation, e.g., the evolution of different cell types within a multicellular organism. Considering a broader landscape for adaptation may reveal obstacles or constraints that limit the evolution of complex multicellularity. Here, we consider an abiotic stress as the main driver of selection and study the evolutionary paths between unicellularity and a life cycle featuring multicellular stages with differentiation. We use mathematical modeling and simulations to show that life cycles with primitive forms of differentiated multicellularity can evolve from a unicellular ancestor in response to a single abiotic stress; however, these forms of differentiated multicellularity are not permanent or guaranteed. Even when populations are exposed to a constant selective pressure and all mutations are beneficial, complex traits can be gained and lost adaptively, as populations continually improve their fitness. Thus, we find that forms of differentiated multicellularity can evolve as a response to abiotic stress, but stress alone is not enough to stabilize a transition toward increased complexity.

One of the major findings of our study concerns the adaptive gains and losses of complexity. In our model cells experience regular bouts of abiotic stress. We observed gains in complexity because cells can improve their survival to the stress by either differentiating into stress-tolerant phenotypes or forming multicellular groups that offer protection. We observed losses of complexity because populations also experienced periodic bouts of no stress, where the only selective driver is to grow as fast as possible. While all populations benefit from mutations that increase growth rate, the strictly unicellular life cycle benefits the most because its cells do not spend time transitioning between forms, i.e., switching between unicellularity and differentiation/multicellularity. Once the growth rate is sufficiently high, the benefit of increased survival is not worth the cost of lost growth and so populations revert to unicellular forms. Consequently, the relative benefit of complex phenotypes vs. unicellularity changes with the genetic background of the population, which means that populations can repeatedly gain and lose complexity in otherwise constant environments.

We expect these adaptive losses in complexity to be widely applicable to populations experiencing abiotic stress. For example, if the abiotic stress is an antibiotic then unicellular populations may be able to survive this stress by forming multicellular biofilms or differentiating into persistence states. If the population later evolves resistance to the stress—or alternatively the antibiotic is not particularly effective—then the relative benefits of evolving differentiation or multicellularity may not be worth their costs, driving populations to be unicellular. Whether such reversions occur will likely depend on a variety of factors including the costs of complexity, the genetic/phenotypic background of cells, and the limits to which populations can adapt in response to the stress. If, for example, the lag times for switching between phenotypes impose a harsh limitation then the accumulated benefits of increasing growth rate (73) may lead populations to revert to a strictly unicellular life cycle. Since there are fundamental biological limitations in the initiation and elongation rates of transcription and translation, it is not possible to switch phenotypes via changed gene expression and protein production faster than on the scale of minutes, which can be long enough for an antibiotic to kill most of the population (74). However, if populations can evolve ways of reducing the costs of complexity, e.g., the lag times for switching between phenotypes, then we expect complexity to be maintained. In this regard, unicellular organisms often use small molecule second messengers, including different nucleotides, Ca^2+^ and NO, to quickly respond to stress without needing to alter gene expression (75–79). Interestingly similar second messengers are also used as signals to form multicellular groups (80), including biofilm formation, as well as to initiate sporulation (75, 81); and they may have even been co-opted for the development of multicellular complexity (80, 82, 83).

Though we found that a single selective pressure could drive the evolution of life cycles with differentiated multicellularity, whether it did so or not depended on two key factors: the initial phenotypic background and the order of mutations. The initial phenotypic background set the costs for forming multicellular groups and differentiating into protected types as well as the death/growth rates in the presence/absence of the abiotic stress. Thus, the initial phenotypic background determines how far a population is from a boundary where it is advantageous to change states, i.e., gain/lose differentiation and gain/lose multicellularity. From a given starting point, mutations that lowered the costs of multicellularity or differentiation acted to maintain forms of complexity or lead to further increases (i.e., evolving differentiated multicellularity), and mutations that increased growth rate or survival acted to promote reversions to a strict unicellular life cycle. The order of mutations also affected the evolution of complexity. In particular we found that early mutations could lock populations into expressing complex traits, i.e., differentiation or multicellularity, and reduce the likelihood of reversions (Figs. 2–4). If neutral mutations could fix, then we found that differentiation and multicellularity more frequently, evolved and reversions to strict unicellularity were less likely. This occurred because neutral mutations allowed unicellular populations to modify traits that increased the relative fitness of differentiation/multicellularity.

By studying the evolution of differentiation and multicellularity together within the same framework we were able to uncover ways in which these two types of complexity interfere with one another, inhibiting the evolution of forms of differentiated multicellularity. One such example was in regard to the evolution of differentiation. Mutations that lowered the time to switch between the stress-resistant phenotype and the growth phenotype favored the evolution of differentiated multicellularity $M_{D}$ vs. multicellularity M. In a framework where M and $M_{D}$ are the only possibilities this would indicate that lowering the time to switch between phenotypes would lead to differentiated multicellularity. Yet, in another pairwise framework where $U_{D}$ is compared with $M_{D}$, the same type of mutation would lead to different conclusions. Lowering the cost to differentiation reduces the relative benefit of forming groups and favors unicellularity over multicellularity, causing $U_{D}$ to be favored over $M_{D}$. These results highlight that relying only on pairwise comparisons can constrain the evolutionary possibilities and miss important ways in which types of complexity interact.

In this study, we considered a type of multicellularity in which groups form via aggregation, but there is another type of multicellularity in which cells remain in groups, called clonal multicellularity. Comparisons of these two types suggest that clonal multicellularity may be better at gaining additional adaptations including more complex traits (64, 84, 85). In line with these findings, the majority of examples of large and complex multicellularity develop clonally (5, 13, 65, 86), yet there are also examples of aggregative multicellularity that have also evolved greater complexity such as cell differentiation and intricate multicellular structures (87–90). The differences between these two types of group formation can sometimes blur as some multicellular organisms use both together (16) and small changes to how these are incorporated in a life cycle can have large adaptive consequences (91). For our study, we focused on aggregative multicellularity because it is typically found in systems that experience environmental fluctuations similar to those imposed by many abiotic stresses. By alternating between unicellular and multicellular forms, aggregative multicellularity can capitalize on the relative advantages in the different environments. In contrast, clonal multicellularity can offer survival benefits in response to stress, but it is at a disadvantage when the stress is removed and populations compete for rapid growth (64). Since cells remain in groups in clonal multicellularity, there are more opportunities for the evolution of traits whose trade-offs limit reversion to unicellularity (92, 93), e.g., those that lead to the creation of specialized cell types that do not actively contribute to group reproduction (94–98). We expect that distinctions between how groups are formed and how life cycles are implemented may significantly affect the role of abiotic stress—or other selective drivers for multicellularity—in shaping the path to further complexity and would be an interesting area for future study.

To constrain the parameter space of our model we made assumptions concerning the structure of the abiotic stress and the population’s response, but there are variations on these assumptions that could alter the results. For example, we assumed the abiotic stress occurred regularly and was detectable so that cells had sufficient time to benefit from implementing strategies such as differentiation or multicellularity. There are other types of abiotic stress, such as variation in access to nutrients, that are severe and unpredictable which might make it difficult for cells to respond via differentiated multicellularity (99–101). In these selective environments populations may evolve heterogeneous strategies such as bet hedging, e.g., in the social amoeba *Dictyostelium discoideum* (102) and the bacteria *Myxococcus xanthus* (103) a fraction of the population remains as single cells (loners and peripheral rods) while others aggregate in response to an altered environment. Instead of switching between unicellularity and multicellularity, populations may also adopt mixed strategies in which multiple life cycles are maintained simultaneously in a population (58, 104, 105). Here mathematical models can be particularly useful in elucidating how the specific structure of the abiotic stress may shape the ecoevolution of unicellular/multicellular populations.

In this paper, we considered abiotic stress whereby the selective driver does not change in response to the evolving populations. We found that while abiotic stress has the potential to select for more complex forms of multicellularity, there are often reversions arising from adaptations that mitigate the severity of the stress. Another possible type of selective driver for multicellularity may be biotic in origin which would then have the potential to coevolve (42). Examples of such drivers include predation or parasitism which both have been found to lead to the evolution of types of complexity (41) including multicellularity (6, 9)—though we note that in the case of multicellularity the predators were specifically prevented from coevolving with the prey. It is unclear whether the coevolutionary process will have any effect on the evolution of complexity. For instance, if a prey evolves to be multicellular in order to escape predation, the predator may adapt to specifically target the multicellular prey thereby selecting either for reversion of the prey back to unicellularity or alternatively increased multicellular size. Distinguishing between these possibilities can help determine whether any selective drivers of multicellularity are more likely to result in the evolution of further complexity or if the process is inherently dominated by chance events.

## Materials and Methods

### Equations for Population Growth.

In order to assess the performance of each life cycle, we define fitness in terms of population growth. Fitness can be derived analytically under the assumption of exponential growth and death, and since the environment switches periodically between $E_{0}$ and $E_{A}$, it is sufficient to do the calculations over one such period. Starting with unicellularity U, we get

[5] $$\begin{array}{c} U=e^{bt_{0}-dt_{A}}, \end{array}$$

as the equation describing population fitness, where $t_{0}$ is the time spent in $E_{0}$ and $t_{A}$ is the time spent in $E_{A}$.

Fitness for differentiated unicellularity, $U_{D}$, is calculated in a similar way as for U, except for a difference in the response to stress. We assume complete differentiation, meaning that when cells are in the survival state, there is no death in $E_{A}$. However, since cells in this survival state cannot reproduce, there is a time lag $\tau_{D}$ when the environment switches back to $E_{0}$ and cells must return to the growth state. During this lag cells do not reproduce, and so they miss an opportunity for population growth. Taken together, the fitness is then

[6] $$\begin{array}{c} U_{D}=e^{b\left(t_{0}-\tau_{D}\right)-d\tau_{D}}, \end{array}$$

where the term $e^{b(t_{0}-\tau_{D})}$ represents the growth in $E_{0}$ and the term $e^{-d\tau_{D}}$ is the death that happens in $E_{A}$ before the cells have differentiated.

For the multicellular life cycle, M, we need to consider the dynamics during the group phase in $E_{A}$. As the outer cells of groups die and get replaced, the inner pool of cells will eventually be emptied. This happens at some time $t^{*}$ in $E_{A}$, and after this, the surface structure starts to collapse. Whether the time spent as a group in $E_{A}$ has passed $t^{*}$ affects the fitness, so we get two different expressions. We formulate them as

[7] $$\begin{array}{c} \left\{\begin{array}{c} M_{1}=e^{b\left(t_{0}-\tau_{G}\right)-d\tau_{G}}\frac{(1-d\left(t_{A}-\tau_{G}\right))+R}{1+R},\;\left(t_{A}-\tau_{G}\right)<t^{*}\\ M_{2}=e^{b\left(t_{0}-\tau_{G}\right)-dt_{A}}\frac{e^{R}}{1+R},\;\left(t_{A}-\tau_{G}\right)>t^{*}, \end{array}\right. \end{array}$$

where the parameter R is the initial ratio of inner to outer cells in a group. The value of R serves as a measure of how well groups protect cells (see *SI Appendix*, *Derivation of Undifferentiated Multicellularity* for a full derivation). The time delay $\tau_{G}$ represents the mismatch of life cycle stage to the environment, which happens when cells switch between single-celled and group states.

For the $M_{D}$ life cycle, we assume that differentiation precedes group formation in $E_{A}$, and multicellularity is lost before switching back to growth in $E_{0}$. We note that other possibilities for the order of events exist, but many produce interference between differentiation and multicellularity (see *SI Appendix*, *Derivation of Differentiated Multicellularity* for a review of the possible series of events). If we assume that groups are completely protected from stress and inner cells have dedifferentiated so they can start to grow earlier in $E_{0}$, we can formulate an expression for fitness:

[8] $$\begin{array}{c} M_{D}=\frac{Re^{b\tau_{D}}+1}{1+R}e^{b\left(t_{0}-\tau_{G}-\tau_{D}\right)-d\tau_{D}}. \end{array}$$

Similar to M, R is the ratio of inner to outer cells in groups. The fitness equations derived here can be used to make comparisons between the life cycles; see *SI Appendix*, *Pairwise Comparisons*.

### Evolutionary Simulations.

We study the evolution of complexity in populations by simulating adaptive trajectories in which populations randomly fix beneficial mutations. Each simulation follows an iterative process in which mutations are chosen randomly from a uniform distribution. The mutations can alter the birth or death rates, b or d; the fraction of time used to switch between types, $T_{D}=\tau_{D}/t_{A}$ or $T_{G}=\tau_{G}/t_{0}$; or whether cells can differentiate or form multicellular groups. Initially, we only consider beneficial mutations though we later expand to consider neutral mutations as well. Mutations that affect rates or fractions, e.g., b or $T_{G}$, change the value by 5%. Each mutation fixes with a probability based on the amount by which it increases relative fitness, denoted as $f_{\mathrm{rel}}$, between the mutant and the resident types. We model the fixation probability to scale linearly with $f_{\mathrm{rel}}$ using the function:

[9] $$\begin{array}{c} P\left(f_{\mathrm{rel}}\right)=p_{\mathrm{low}}+\frac{p_{\mathrm{high}}-p_{\mathrm{low}}}{f_{\max}-f_{\min}}\left(f_{\mathrm{rel}}-f_{\min}\right). \end{array}$$

This implies that we accept all mutations with $f_{\mathrm{rel}}>f_{\max}$ with the probability $p_{\mathrm{high}}$ and discard all mutations with $f_{\mathrm{rel}}<f_{\min}$. Mutations within $f_{\min}<f_{\mathrm{rel}}<f_{\max}$ are accepted with a probability p in the range $\left[p_{\mathrm{low}},p_{\mathrm{high}}\right]$. Specifically, the values used in the simulations are $f_{\min}=1$, $f_{\max}=1.5$, $p_{\mathrm{low}}=0$, and $p_{\mathrm{high}}=1$. This means that mutations resulting in a fitness benefit of more than 50% are always accepted, and all mutations must be beneficial to have a chance of fixing. For simplicity, we assume that beneficial mutations are rare, and so new mutations always appear in homogeneous populations.

Each simulation starts as a unicellular U population. We follow the evolution of each population until 50 mutations have fixed and repeat the process for 10,000 independent runs. The initial values of the time delays are shown in *SI Appendix*, Fig. S7 and given in *SI Appendix*, Table S1. Initial values for the other parameters are b=1.1, d=1, $t_{0}=t_{A}=10$, and R=5.

To study the effects of neutral mutations on adaptation, we adjust the parameters for the acceptance function in Eq. **9**. A neutral mutation has no effect on relative fitness, $f_{\mathrm{rel}}=1$, so to allow it a 20% chance of fixing we set $p_{\mathrm{low}}=0.2$. We note that mutations are neutral in regard to the current life cycle, so a mutation that is neutral may be deleterious should the life cycle change. For instance, a mutation in a unicellular population U that increases either of the time delays will be neutral but it would be deleterious in any of the other life cycles: $U_{D}$, M, or $M_{D}$.

## Supplementary Material

Appendix 01 (PDF)

## Data Availability

There are no data underlying this work.
