# Supplementary material for: Adaptive evolutionary trajectories in complexity: Transitions between unicellularity and facultative differentiated multicellularity
Source: Proc Natl Acad Sci U S A. 2025 Jan 22;122(4):e2411692122. doi: 10.1073/pnas.2411692122 (PMC11789074; doi:10.1073/pnas.2411692122)
Supplement: Supplementary file 1 — Appendix 01 (PDF) [file pnas.2411692122.sapp.pdf]

Supporting information

Sensitivity analysis for the environmental duration. Here, we consider how our results are affected by varying the time spent in each environment (t_0 and t_A respectively). The top panels of Fig. S1 show that the time spent in E_0 does not significantly change the landscape of which life cycle is fittest. This is because after the time in the E_0 environment exceeds a delay (either for group formation or switching phenotypes), all life cycles grow at the same rate. For example, after τ_D the U_D population has de-differentiated back to its growth phenotype and its dynamics are identical to a U population that never differentiated. The relative fitness in E_0 then is entirely determined by the costs associated with switching phenotypes and these only occur for the initial τ_D amount of time and not the total additional time spent in E_0.

Panels D)-F) in Fig. S1 show that varying t_A does affect which life cycle has the highest fitness. This effect comes from the different death rates experienced by the life cycles in E_A. After the initial time delay for differentiation, life cycles with differentiation (U_D and M_D) are completely protected while the U and M populations continue to decay as t_A increases. Thus, the relative benefit of differentiation compared to these other life cycles increases as t_A increases.

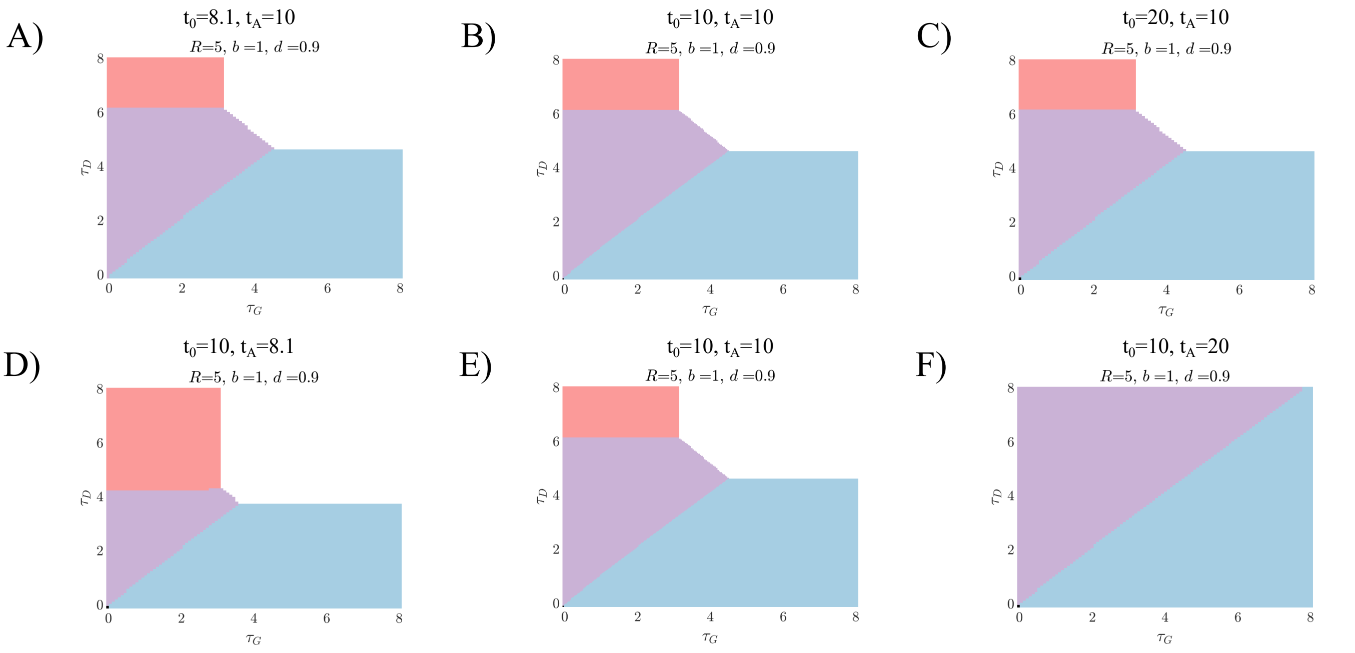

Fig. S1. Sensitivity analyses for the time spent in E_0 and E_A environments. In the main text, we assumed that the stressful environment (E_A) has the same duration as the non-stressful environment (E_0), i.e. we set $t_A = t_0$. To explore how this assumption affects the results, we have recreated Fig 4C for different values of t_0 and t_A. A)-C) Each plot is similar to Fig 4C and shows which life cycle is fittest in a parameter space of the time delays for group formation τ_G and differentiation τ_D. We kept the value of t_A fixed and varied the value of t_0 so that it is smaller than t_A (A), equal to it (B), and larger than it (C). These panels show that the time spent in E_0 does not have any qualitative effect on the results. D)-F) In these panels, we fixed t_0 and varied the time in the stressful environment t_a so that it is smaller than t_0 (D), equal to it (E), and larger than it (F). These panels show that the time spent in E_A has a significant effect particularly in (F) where U and M are no longer the fittest life cycles for any combination of time delays in this parameter space.

Derivation of undifferentiatied multicellularity. In the undifferentiated multicellular lifestyle, M, cells grow as single cells in E_0 but can improve their fitness by aggregating into groups when the stress is present, E_A. We assume that groups are tightly packed so that there is no space for cell reproduction in the group phase, and that groups are spherical and of equal size. Moreover we assume that it takes some time τ_G to form and dissociate groups, so when entering E_A the cells initially die with the rate d and when leaving E_A they are temporally prevented from growth, see Fig. S2.

In a group, cells can be divided into two classes: the outer cells that make up the surface of the group and the inner cells. Cells inside a group are physically protected from the stress, while cells on the group surface die with the rate d. When a cell on the surface dies it immediately gets replaced by a cell from the inside. This divides the population growth of M into two cases depending on whether groups have any inner cells left when leaving E_A. Growth of the M lifestyle can be described as

$$\begin{cases} M_1 = e^{b(t_0-\tau_G)-d\tau_G} g_1(t_A - \tau_G) \\ M_2 = e^{b(t_0-\tau_G)-d\tau_G} g_1(t^*) g_2(t_A - \tau_G - t^*), \end{cases} \quad [10]$$

where M_1 represents the case where groups still have inner cells left at the end of the stress phase, and M_2 is the case when there are no inner cells left. The functions g_1 and g_2 describe the change in population size during E_A and t^* is the time it takes to empty the inner cell pool.

The functions g_1 and g_2 can be determined by modeling the dynamics of the inner and outer cells with a set of differential equations. Starting with the first case, M_1, we can represent the dynamics for inner ($M_{1,i}(t)$) and outer ($M_{1,o}(t)$) as

$$\begin{cases} \frac{M_{1,o}(t)}{dt} = 0 \\ \frac{M_{1,i}(t)}{dt} = -dM_{1,o}(t). \end{cases} \quad [11]$$

Integrating this gives

$$\begin{cases} M_{1,o}(t) = M_{1,o}(0) \\ M_{1,i}(t) = -dM_{1,o}(0)t + M_{1,i}(0) \end{cases} \quad [12]$$

where $M_{1,i}(0)$ and $M_{1,o}(0)$ are the initial amounts of inner and outer cells. If $g_1(t)$ is the change in population size during t_A it can be expressed as

$$g_1(t) = \frac{M_{1,o}(0)(1-dt) + M_{1,i}(0)}{M_{1,o}(0) + M_{1,i}(0)}. \qquad [13]$$

If we introduce $R = \frac{M_{1,i}(0)}{M_{1,o}(0)}$ as the initial fraction of inner to outer cells and let $t = t_A - \tau_G$ be the time spent as groups in E_A, we get the expression

$$g_1(t_A - \tau_G) = \frac{(1 - d(t_A - \tau_G)) + R}{1 + R}. \qquad [14]$$

Putting this into M_1 in Eq. 10 we get

$$M_1 = e^{b(t_0-\tau_G)-d\tau_G} \frac{(1 - d(t_A - \tau_G)) + R}{1 + R}. \qquad [15]$$

In order to derive M_2 we first need to find an expression for g_2, which represents the loss of outer cells when there are no inner cells left. We start by calculating how much time t^* it takes for the inner cell pool to be emptied. This is done by solving $M_{i,1}(t^*) = 0$ in Eq. 12 and we find that $t^* = R/d$. After this time the population dynamics changes in that the remaining outer cells just die with the rate d with no replacement of inner cells. So, when $t_A > \tau_G + t^*$ the population dynamics is described by

$$\begin{cases} \frac{M_{2,o}(t)}{dt} = -dM_{2,o}(t) \\ \frac{M_{2,i}(t)}{dt} = 0. \end{cases} \qquad [16]$$

By integrating the upper equation we can express the change in population size as

$$g_2(t_A - \tau_G - t^*) = e^{-d(t_A-\tau_G-t^*)}, \qquad [17]$$

where $t_A - \tau_G - t^*$ is the time spent in E_A after the groups have run out of inner cells. Using that $t^* = R/d$ we get

$$g_2(t_A - \tau_G - t^*) = e^{-dt_A+d\tau_G+R} \qquad [18]$$

and putting this into the equation for M_2 together with the result in Eq. 14 we get

$$M_2 = e^{b(t_0-\tau_G)-dt_A} \frac{e^R}{1 + R}. \qquad [19]$$

These calculations show that M_1 and M_2 can be expressed as

$$\begin{cases} M_1 = e^{b(t_0-\tau_G)-d\tau_G} \frac{(1-d(t_A-\tau_G))+R}{1+R} \\ M_2 = e^{b(t_0-\tau_G)-dt_A} \frac{e^R}{1+R}. \end{cases} \qquad [20]$$

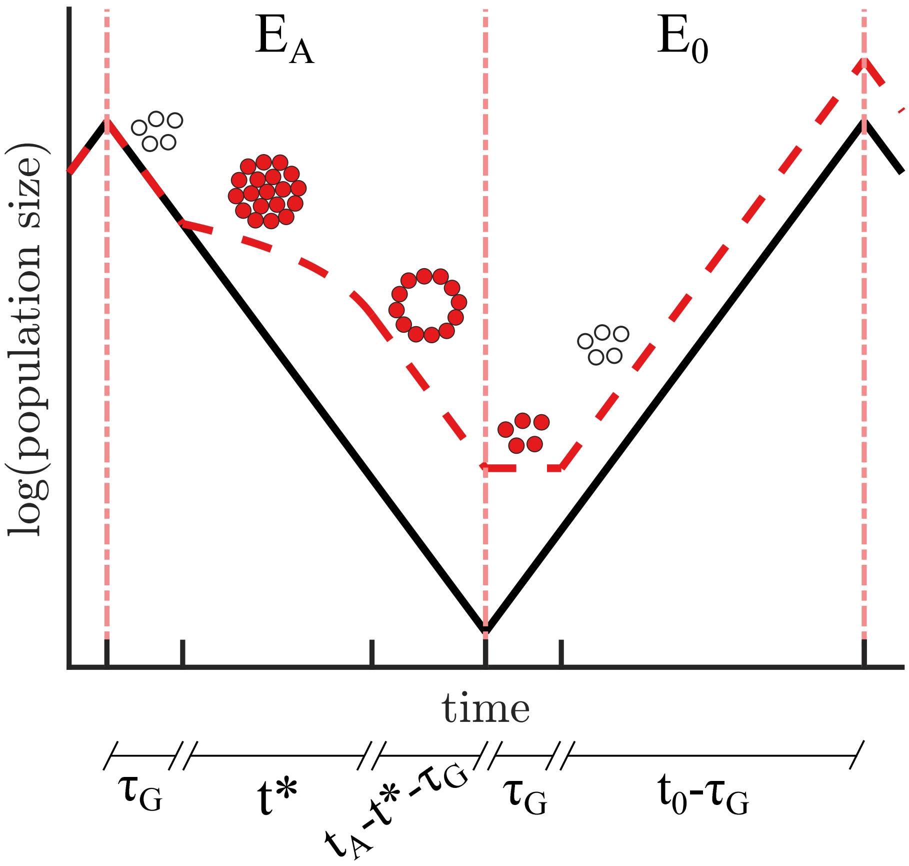

Fig. S2. Multicellular groups provide physical protection during stress. A schematic illustrates the dynamics of two populations (U, solid line and M, dashed line) as the environment switches between E_A and E_0. The U population grows exponentially in E_0 and dies in E_A. The M population acts similar to U in E_0, except that it initially experiences a time lag τ_G where it is prevented from growth. In E_A, M dies at the same rate as U during τ_G when groups are formed. Once the groups are formed the death rate of M is reduced as long as there are inner cells left. When groups have run out of inner cells they die at the same rate as undifferentiated single cells.

Derivation of differentiated multicellularity. Upon formation of differentiated multicellularity (M_D) in E_A the outer cells are resistant to the stress and provide perfect protection for inner cells. Since inner cells are not affected by stress, they can switch back to a growth state while still in E_A. This switching back to the growth state can provide an advantage in E_0 allowing inner cells to grow as soon as multicellular groups have dissociated.

We can derive an expression for the fitness for M_D, depending on the order of differentiation and group formation within the life cycle. Phenotypic switching can happen either sequentially e.g. groups can only form after all cells have switched to survival mode, or in parallel i.e. differentiation and group formation occur at the same time, see Fig. S3. The equations describing the fitness for each of the cases in Fig. S3 can be summarized as

$$M_D = e^{b(t_0-\tau)} f(t), \qquad [21]$$

where the factor $e^{b(t_0-\tau)}$ corresponds to population growth in E_0 after groups have dissociated and cells have switched to growth mode. The function $f(t)$ describes the change in population size during the phenotypic switching and it depends on the life cycle construction. There is no contribution from the population dynamics in E_A once cells have differentiated and formed groups since they would then have full protection from the stress.

If phenotypic switching happens in parallel we let $f(t) = f_{0,D}(\tau_D)f_{0,G}(\tau_G)f_{A,D}(\tau_D)f_{A,G}(\tau_G)$ be a function that is composed of $f_{0,D}(\tau_D)f_{0,G}(\tau_G)$ which corresponds to the population change during phenotypic switching in E_0 and $f_{A,D}(\tau_D)f_{A,G}(\tau_G)$ which corresponds to the population change during phenotypic switching in E_A. For the case where switching between growth and survival modes always happens before group formation (M_{D1}, first row in Fig. S3) we have $f_{A,D} = e^{-d\tau_D}$ and $f_{A,G} = 1$ in E_A since all cells are protected after τ_D. Similarly, in E_0 we have that $f_{0,D} = 1$ and $f_{0,G} = 1$ because even if the inner cells have switched back to growth mode they need to wait until groups have dissociated. The resulting expression for M_{D1} is

$$M_{D1} = e^{b(t_0-\tau_G-\tau_D)-d\tau_D}. \qquad [22]$$

When group formation and dissociation precede switching between growth and survival modes (M_{D2}, second row in Fig. S3) we let $f_{A,G} = e^{-d\tau_G}$ and $f_{A,D} = \frac{1-d\tau_D+R}{1+R}$ in E_A (see Supplementary Information "Derivation of undifferentiated multicellularity" derivations for M). Here, cells die with the rate d during group formation and while switching phenotype there is some death of the group's outer cells. In E_0, the inner cells can start to grow once the groups have dissociated while the outer cells first need to switch mode. Hence, $f_{0,G} = 1$ and $f_{0,D} = \frac{1-(R-d\tau_D)e^{b\tau_D}}{a-d\tau_D+R}$. In particular, $f_{A,D}f_{0,D} = \frac{1+(R-d\tau_D)e^{b\tau_D}}{1+R}$. The final expression for M_{D2} is

$$M_{D2} = e^{b(t_0-\tau_G-\tau_D)-d\tau_G}\frac{1+(R-d\tau_D)e^{b\tau_D}}{1+R}. \qquad [23]$$

The M_{D3} case is when groups form before phenotypic switching in E_A, and in E_0 phenotypic switching happens first (third row in Fig. S3). This means that $f_{A,G} = e^{-d\tau_G}$ because cells die during group formation and $f_{A,D} = \frac{1-d\tau_D+R}{1+R}$ as some outer cells die before they have switched into survival mode. In E_0 we have that $f_{0,G} = 1$ and $f_{0,D} = 1$ because there is no cell growth until the groups are dissociated. The resulting expression for M_{D3} is

$$M_{D3} = e^{b(t_0-\tau_G-\tau_D)-d\tau_G}\frac{(1-d\tau_D)+R}{1+R}. \qquad [24]$$

In the M_{D4} case (fourth row in Fig. S3) cells switch to survival mode in E_A before forming groups so $f_{A,D} = e^{-d\tau_D}$ and $f_{A,G} = 1$ as all cells have full protection during group formation. Upon removal of the stress (E_0) the groups first dissociate so the former inner cells can start to reproduce while the outer cells also need to switch to growth mode. Particularly, this means that $f_{0,G} = 1$ and $f_{0,D} = \frac{1+Re^{b\tau_G}}{1+R}$. To summarize, the growth equation for M_{D4} can be written as

$$M_{D4} = e^{b(t_0-\tau_G-\tau_D)-d\tau_D}\frac{1+Re^{b\tau_D}}{1+R}. \qquad [25]$$

In both of the M_{D5} and M_{D6} cases the phenotypic changes happen in parallel. For M_{D5} (fifth row in Fig. S3) $\tau_G > \tau_D$ so in E_A we have that $f_{A,D} = e^{-d\tau_D}$, while $f_{A,G} = 1$. When the stress is removed cells are prevented from growth until the groups have dissociated so $f_{A,D} = 1$, while $f_{A,G} = 1$. In summary, the total growth of M_{D5} is

$$M_{D5} = e^{b(t_0-\tau_G)-d\tau_D}. \qquad [26]$$

The M_{D6} (sixth row in Fig. S3) is when $\tau_D > \tau_G$. This means that in E_A $f_{A,G} = e^{-d\tau_G}$ and $f_{A,D} = \frac{1-d(\tau_D-\tau_G)+R}{1+R}$ as there will be some death of the outer cells during the switch to survival mode. In E_0 there is no cell reproduction during τ_G so $f_{0,G} = 1$, but after dissociation the inner cells can grow so $f_{0,D} = \frac{1-d(\tau_D-\tau_G)+R}{1+(R-d(\tau_D-\tau_G)))e^{b(\tau_D-\tau_G))}}$. The total equation for fitness of M_{D6} is

$$M_{D6} = e^{b(t_0-\tau_D)-d\tau_G}\frac{1+(R-d\Delta\tau)e^{b\Delta\tau}}{1+R}, \qquad [27]$$

where $\Delta\tau = \tau_D - \tau_G$ in the expression for M_{D6}. The M_{D4} case is the life cycle structure that we use for M_D in the main analysis.

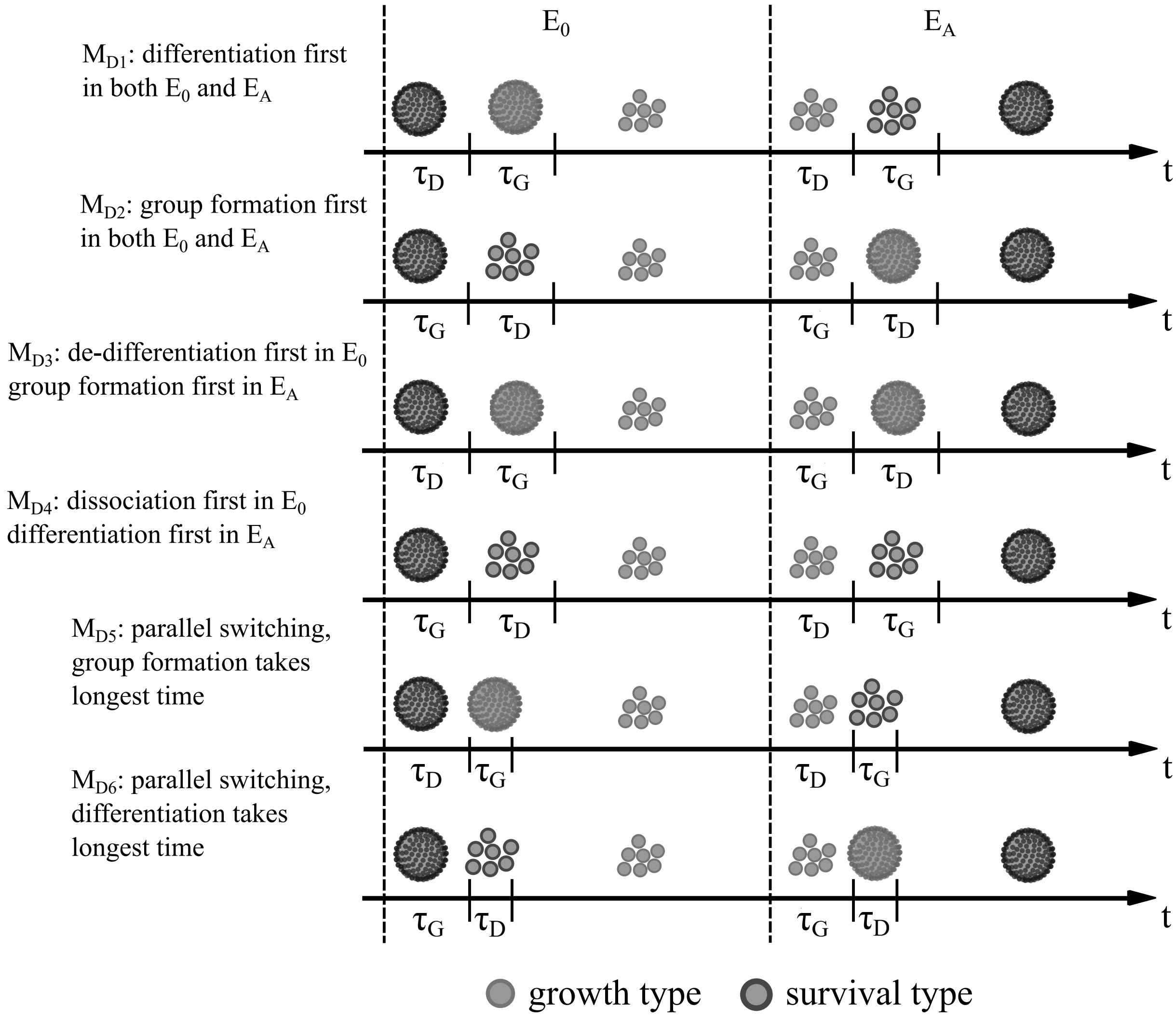

Fig. S3. Effects from the order of phenotypic switches on the M_D life cycle structure. A schematic illustrates how the events, i.e. group formation/dissociation and switching between survival/growth phenotypes, can occur in different orders throughout the M_D life cycle. For all cases M_D enters E_A as undifferentiated single cells and leaves E_A as differentiated multicellular groups. In cases $M_{D1} - M_{D4}$ the events are sequential i.e. one phenotypic switch happens at a time. For M_{D5} and M_{D6} the processes are parallel such that two phenotypic changes can take place simultaneously and the switching finishes faster.

Comparing fitness conditions for U_D vs M_D. The fitness comparison between U_D and M_D depends on three variables (τ_G, τ_D and b) so the there are several ways to construct a parameter space, see Fig. S4. From the figure we see that once populations are in a place where U_D is fittest it is not possible to evolve to a place where M_D is favored, assuming that only beneficial mutations are allowed. The reason is that the mutations required to evolve M_D are neutral to U_D so there is no selection for them to fix. In contrast, beneficial mutations in the M_D lifestyle, especially mutations in τ_D, lead to loss of the multicellular trait. This analysis shows that by only allowing beneficial mutations it is difficult to maintain the M_D life cycle.

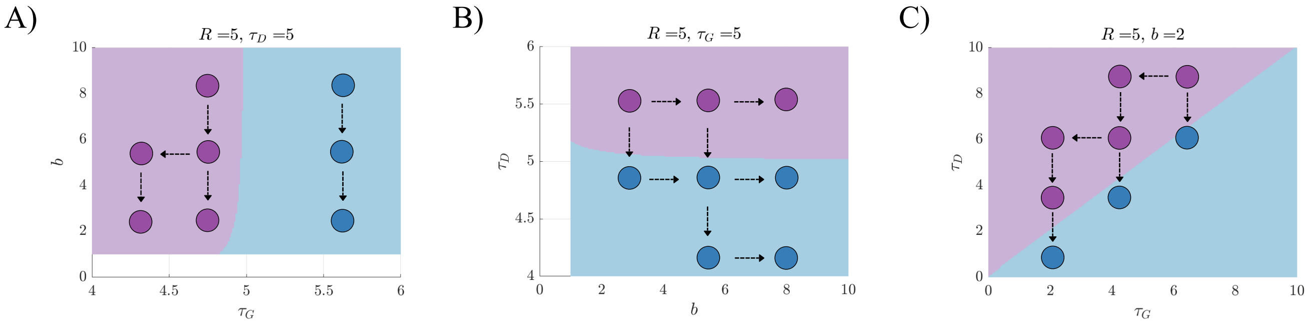

Fig. S4. Beneficial mutations and reversions from M_D to U_D. A) A parameter space shows which of U_D (blue) and M_D (purple) has the highest fitness with respect to the traits τ_G and b. Shown are two examples of adaptive walks in the parameter space where populations are locked into the M_D and U_D life cycles respectively. B) A parameter space shows similar data as A), but for the traits b and τ_D. Sample trajectories show that mutations in b mostly preserve the current life cycle while mutations in τ_D make M_D revert to U_D. C) A similar parameter space as A) and B) but for the trait values of τ_G and τ_D. Sample trajectories show that populations can maintain the M_D life cycle if mutations occur in τ_G, but mutations in τ_D will lead to reversion to U_D.

Comparing fitness conditions for M vs M_D. There are five parameters involved in comparing the fitness of M vs the fitness of M_D: R, t_A, b, d, and τ_D. If we assume that R and t_A are not evolvable e.g. they are environmental variables, this leaves three parameters. The results from the comparisons are shown in Fig. S5, where the parameter space in panel A) shows that beneficial mutations in b and τ_D will change whether differentiation is fitter or not, except when b is very low. Adaptation in b affects M and M_D similarly so it does not change which is fitter. For the differentiation time delay, lower values of τ_D make M_D fitter, but mutations in τ_D are adaptive only in M_D, because they are neutral in M. The parameter space in B) shows that improving survival to the stress, i.e. decreasing d, will eventually lead to loss of differentiation regardless of the value of τ_D. Beneficial mutations in τ_D will lead to an area of parameter space where M_D is fitter as long as the death rate d is above a certain threshold $d \approx 0.32$ here. Similar to the result in Fig. S4, the comparison of M and M_D shows that differentiated multicellularity is difficult to maintain when all mutations are beneficial.

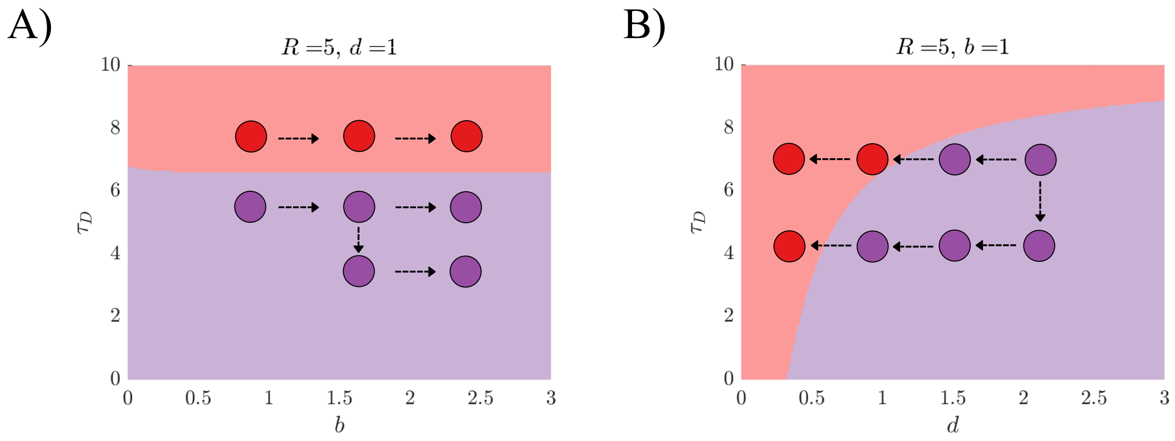

Fig. S5. Beneficial mutations and reversions from M_D to M. A) A parameter space shows which life cycle M (red) or M_D (purple) is fitter for different combinations of traits b and τ_D. Sample adaptive walks show that beneficial mutations in this parameter space rarely lead to transitions between the red M and purple M_D regions. B) A similar plot as in A) is shown but for the traits d and τ_D. Here, the example adaptive walks show that trajectories stay in the M_D region for mutations in τ_D, but mutations that reduce d will always lead trajectories into regions that only favor M.

Sensitivity analysis of the group parameter R. Here, we explore the effects from varying values of R, see Fig. S6. For low R values a small fraction of cells get protection, so the benefits from forming groups are small. Therefore, M is never the fittest life cycle for $R \leq 1$. Higher R values represent group configurations that offer protection for a larger fraction of cells, so e.g. $M = 10$ multicellularity has higher fitness for a larger set of parameter values. As R increases M is fitter than M_D for a larger set of parameter values. The reason is that M pays lower costs (only τ_G) and the benefits from forming groups are high.

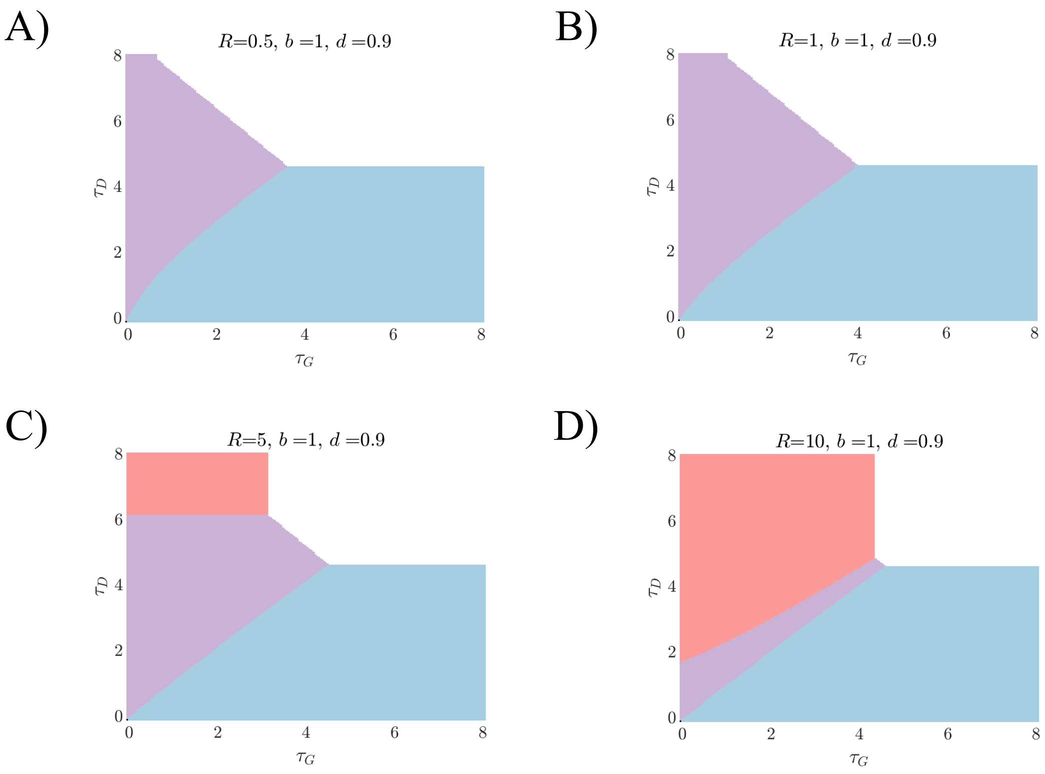

Fig. S6. Sensitivity analysis for different group sizes/organizations. The fitness of life cycles with a multicellular stage may depend on the structure of multicellular group, in particular how large a fraction (R) of the cells are protected inside the group from the stress. Here, Fig 4C is recreated for varying values of the group-size parameter R: A) shows $R = 0.5$, where more cells are exposed to the stress than protected; B) shows $R = 1$ where the same number are exposed as protected; C) shows $R = 5$ which is same as used in the main text; and D) shows $R = 10$ in which many more cells are protected than exposed. In A) and B) multicellularity alone is never the fittest while in D) this region is much larger than the differentiated multicellularity region.

Sensitivity to initial conditions. For our simulations we selected initial conditions spread across parameter space that still allowed for adaptation (shown in Fig. S7 and listed in Table S1). We ran simulations for each set of initial conditions and tracked the life cycle (U, U_D, M, or M_D) in time. (see Fig. S8). We find that the initial condition largely dictates the evolutionary trajectory. In cases such as A) and J) the U life cycle occurs exclusively, because simulations that start in the U region cannot gain beneficial mutations in τ_D and τ_G which are needed to transition to regions that favor M or U_D. The initial conditions used in Fig. 5 are A), C), F), and I) for panels A), B), E), and G) respectively.

Initial position	T_G	T_D
A)	0.5	0.5
B)	0.5	0.4
C)	0.5	0.3
D)	0.3	0.7
E)	0.2	0.7
F)	0.1	0.7
G)	0.3	0.45
H)	0.3	0.35
I)	0.2	0.5
J)	0.2	0.4

Table S1. Initial parameter values used for evolutionary simulations. Presented are the initial values of τ_G and τ_D (here normalized with the time spent in each environment) used for the evolutionary simulations.

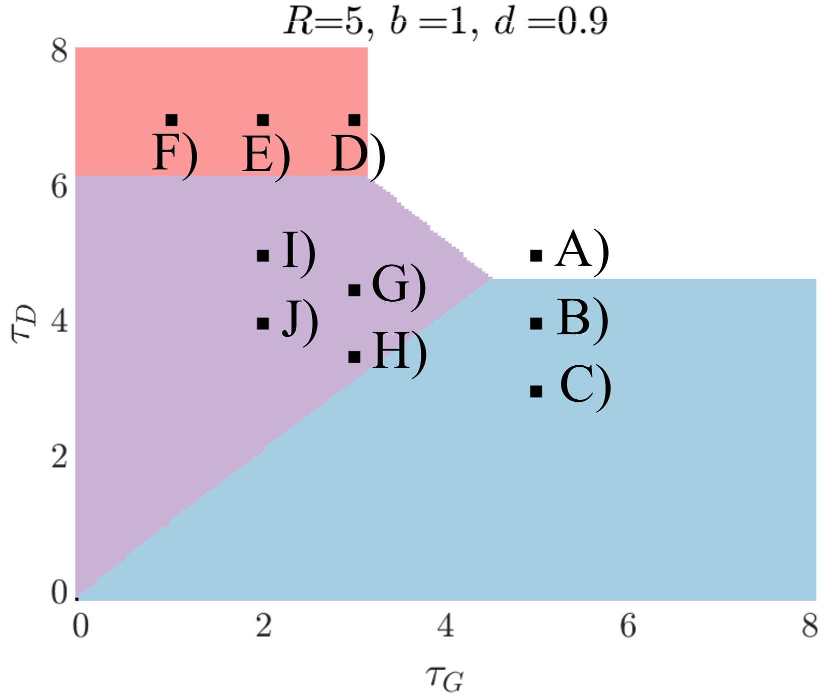

Fig. S7. Initial parameter settings used for simulations. A set of initial conditions used for our simulations are shown in a parameter space. We use initial conditions from all four regions where each of the lifestyles has the highest fitness.

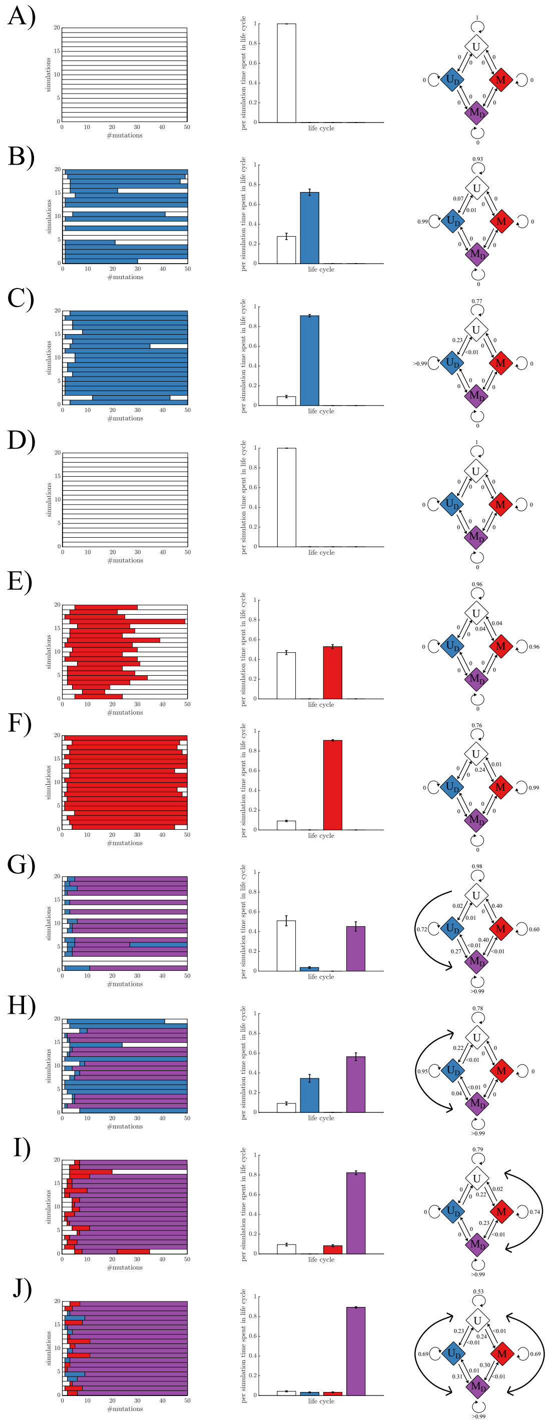

Fig. S8. Initial parameter settings affects evolutionary transitions. A) A plot (left) shows a sample from simulations run for the initial condition A) in Fig. S7. The histogram (middle) shows the fraction of time during the simulations that is spent in each of the lifestyles. The diagram (right) shows the transition probabilities between the lifestyles. Panels B)-J) show similar data as panel A) but for the initial conditions B)-J) in Fig. S7.

Neutral mutations. In the majority of our study we consider the fixation of beneficial mutations, i.e. those that increased fitness. Here, we allow mutations that are neutral to fix with a 20% probability. We note that neutral mutations do not change the fitness of the current life cycle but may have effects on fitness if the population evolves a different life cycle. An example of a neutral mutation is if a time delay in either differentiation or group formation changes in a U population. Allowing neutral mutations to fix opens up new evolutionary trajectories for each initial parameter setting, see Fig. S9. Neutral mutations also lead to higher frequency of gained complexity and less reversions, see Fig S10.

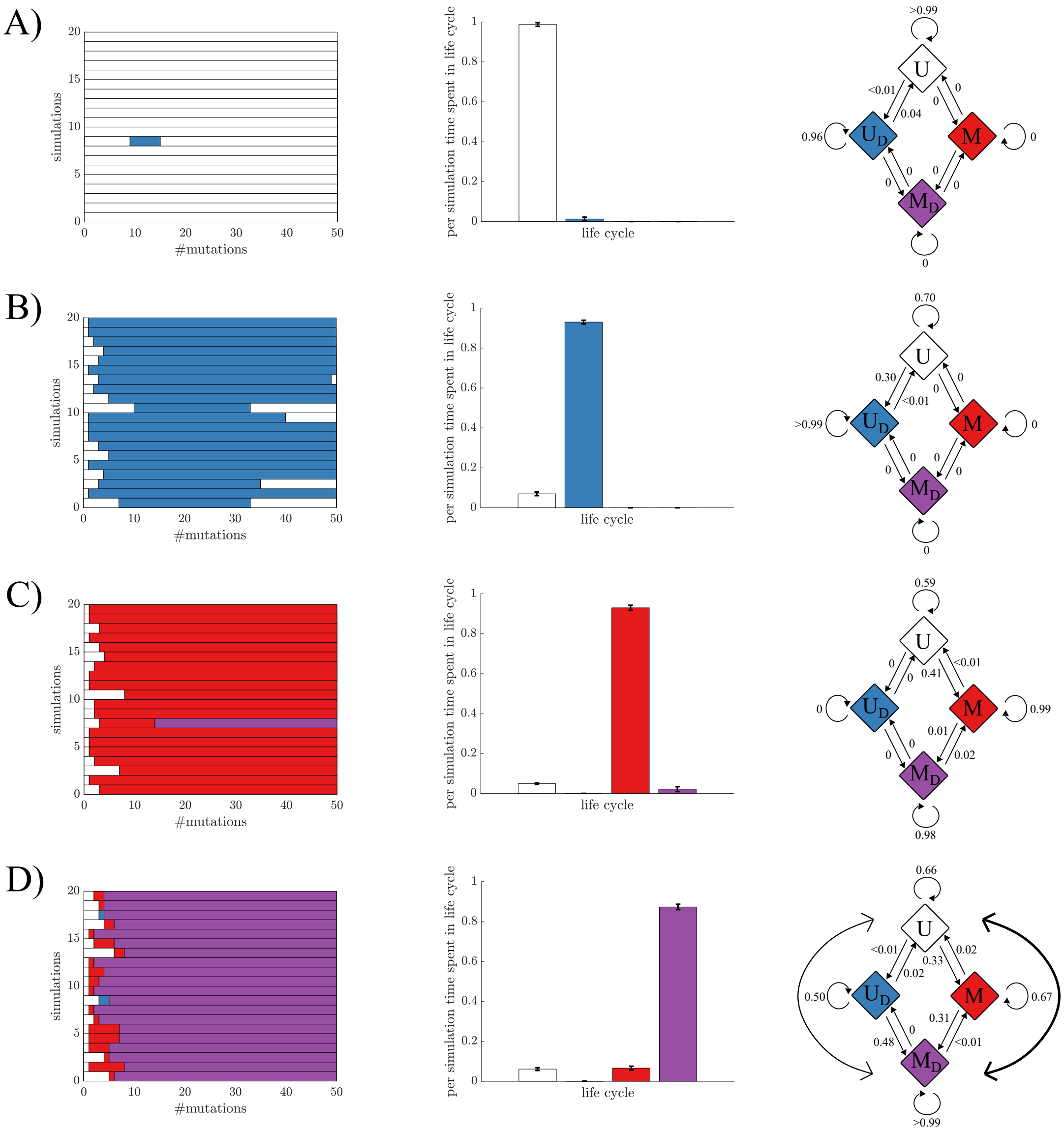

Fig. S9. Neutral mutations and increased diversity of evolutionary routes. Neutral mutations are allowed with 20% probability, which opens up for evolution of new life cycles. A) A plot (left) shows a sample from simulations run for the initial condition A) in Fig. S7 and here U_D is able to evolve with low frequencies. The histogram (middle) shows the fraction of time during the simulations that is spent in each of the lifestyles. The diagram (right) shows the transition probabilities between the lifestyles. B) The panels show similar data as A) but for the initial condition C) in Fig. S7. Since U_D is already dominant in this setting, neutral mutations do not have a big impact on evolution. C) The panels show similar data as A) but for the initial condition F) in Fig. S7. Allowing neutral mutations enables the M_D lifestyle to evolve in this setting. D) The panels show similar data as A) but for the initial condition I) in Fig. S7. The neutral mutations open up for new evolutionary routes from U to M_D, in this case from U to U_D to M_D.

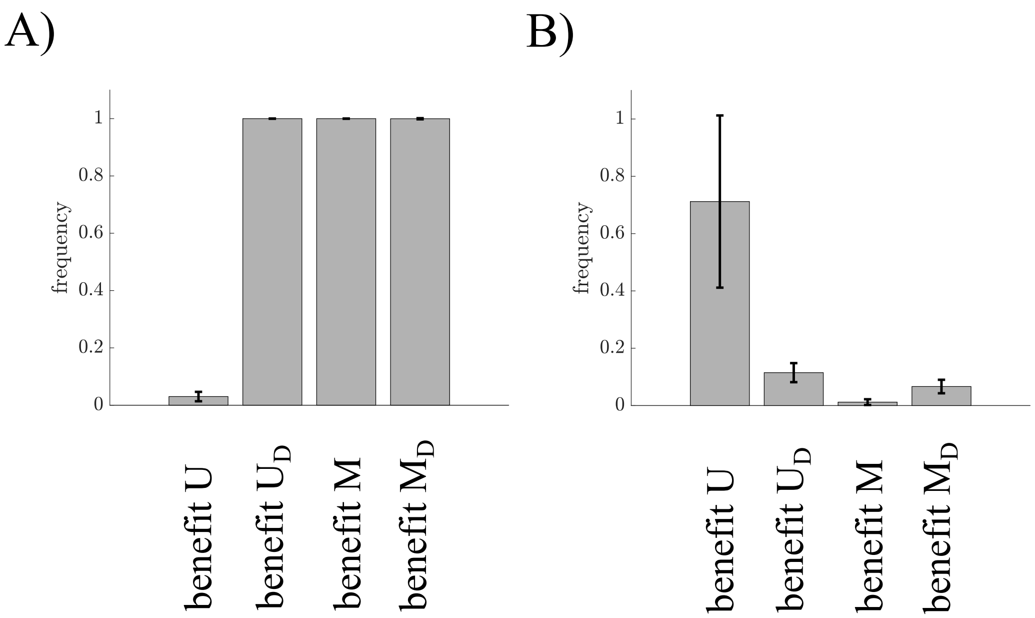

Fig. S10. Neutral mutations enhance evolution of complex lifestyles. A) A histogram shows the frequency of evolved complex traits when neutral mutations are allowed. Compared to Fig. 6A) complex traits evolve to a larger extent when neutral mutations are allowed. B) A histogram shows the frequency of reversions back to U given that complexity first evolved. We observe that reversions are more rare than when mutations are only beneficial, see Fig. 6B). Here, U is an exception because for beneficial mutations this case did not evolve complex traits at all.

Alternative evolutionary trajectories. The routes from U to M_D can be altered by varying the initial parameter settings. By starting the simulations with trait values close to the U_D boundary, such as case H) in Fig. S7, the evolutionary trajectory goes from U to U_D to M_D. For a different starting point we find that evolution takes other paths. Using case J) in Fig. S7 as the initial state, M_D can be reached via either of U_D first or M first.

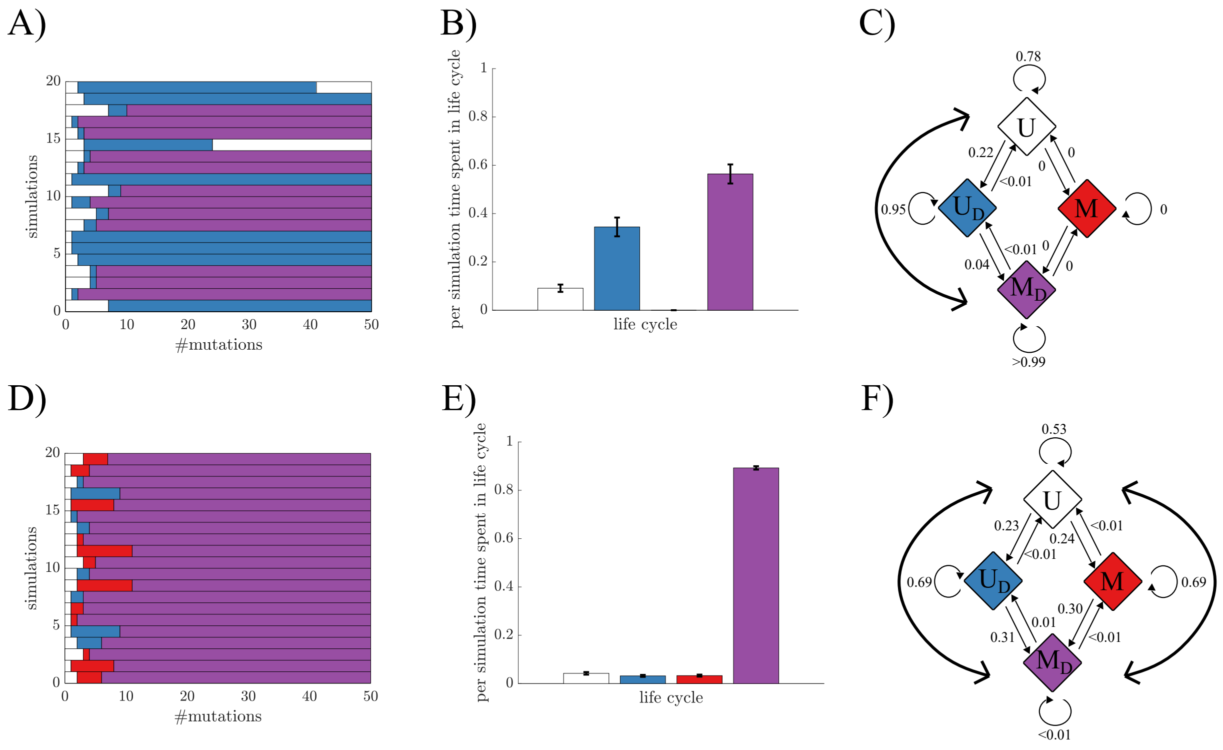

Fig. S11. Evolutionary trajectories take different routes towards differentiated multicellularity. A) A plot shows a sample from simulations where H) in Fig. S7 was used as the initial condition. The data shows that M_D evolves via a transition to U_D. B) A histogram shows the fraction of time spent in each lifestyle, and here M_D is the most common. C) A diagram shows the transition probabilities between the lifestyles. For the initial condition H) the route between U and U_D goes via U_D. D)-F) Similar data is shown as in A)-C) but for the initial condition J). In this case evolution can take either of the routes i.e. differentiation first or multicellularity first, from U to U_D.

Pairwise comparisons. The relative fitness of life cycles can be analyzed by doing pairwise comparisons. Here we derive analytical expressions for the conditions when one life cycle is fitter than another.

Differentiated unicellularity vs undifferentiated unicellularity First we derive a condition where differentiated unicellularity U_D is fitter than undifferentiated unicellularity U. Taking the logarithms of the growth equations (Eq. 5 and Eq. 6) and setting $U_D > U$ we get

$$bt_0 - b\tau_D - d\tau_D > bt_0 - dt_A. \quad [28]$$

Dividing by d and t_A we can write the expression in a dimensionless form. If we define the dimensionless parameters as

$$\begin{cases} T_D = \frac{\tau_D}{t_A} \\ r = \frac{b}{d}. \end{cases} \quad [29]$$

and solve for T_D the resulting condition for when U_D has higher fitness than U is

$$T_D < \frac{1}{1+r}. \quad [30]$$

Undifferentiated multicellularity vs undifferentiated unicellularity With a similar approach we can calculate when undifferentiated multicellularity M is fitter than undifferentiated unicellularity U. For M there are two cases depending on whether any inner cells are left (see Supplementary Information "Derivation of undifferentiated multicellularity"). Here, we assume that the stress is around long enough for groups to run out of inner cells. Using Eq. 5 and Eq. 7 and taking the logarithm of $U < M$ we get

$$bt_0 - dt_A < bt_0 - b\tau_{G2} - d\tau_{G1} - dt_A + d\tau_{G1} + R - ln(1+R). \quad [31]$$

Simplifying this gives

$$S > b\tau_{G2}, \quad [32]$$

where $S = R - \ln(1+R)$ is the shape parameter that depends on the fraction of inner to outer cells ($R = M_i(0)/M_o(0)$).

Differentiated unicellularity vs differentiated multicellularity For a comparison between U_D and differentiated multicellularity (M_D) we use Eq. 6 and Eq. 8. Taking $U_D < M_D$ and rearranging terms gives the condition

$$e^{b\tau_D} > \frac{e^{b\tau_G}(R+1) - 1}{R} \quad [33]$$

for when M_D has higher fitness.

Undifferentiated multicellularity vs differentiated multicellularity Comparing under what conditions M_D is fitter than M we use Eq. 8 and Eq. 7 and again make the assumption that M has run out of inner cells when leaving E_A. By setting $M < M_D$ and rearranging terms we get the expression

$$e^{b\tau_D} < \frac{1}{e^{R-d(t_A-\tau_D)} - R} \quad [34]$$

for when M_D has higher fitness than M.

Sensitivity analysis of carrying capacities. In the main analyses, populations experienced unlimited growth in E_0. To explore the effects of density dependence, we let the populations grow together up to a carrying capacity. Additionally, we explicitly modeled the group dynamics during E_A by performing an agent-based simulation. In this simulation, populations grew up to a carrying capacity $c = 10^5$ in E_0 before being transferred into the E_A environment. In E_A, the death of U and M cells was simulated as a stochastic process. Upon entering E_A, there is first a death phase during τ_G where all cells die at the same rate. After τ_G, the M cells are first distributed in groups and get assigned positions as either interior or exterior cells. In addition to the parameter R, which determines the initial proportion of interior to exterior cells, we had to introduce a parameter that determines the number of cells per group. Assuming that all groups, except possibly one, have the same size when formed, we let $M_{G,O} = 20$ represent the initial number of outer cells. The death of U and M cells was simulated by selecting cells for death with a probability p_d at each time step. We set the death probability to $p_d = 0.1$, and the time step was calculated according to

$$\Delta t = \frac{t_A \ln(p_d)}{-p_d(t_A - \tau_G)}$$

to match the dynamics observed from the analytical calculations. If a cell belonging to a group died, an interior cell (if any remained) from that group was assigned as the new exterior cell.

We performed this analysis both for the pairwise comparisons of U versus U_D and U versus M, and for the joint comparison of all four life cycles (see Fig. S12). The results show qualitatively similar outcomes for the fitness comparisons, despite population growth being limited by a carrying capacity. The main difference compared to our analytical results is that some parameter values (e.g., b in the U vs M simulation) had to be slightly modified because the stochastic simulation used integer cell numbers, whereas the analytical calculations could give decimal numbers.

The results from the density-dependence simulations and the analytical calculations are similar because after the initial time delays for group formation or differentiation, all life cycles grow at the same rate in E_0. In practice, introducing a carrying capacity has the same effect as shortening t_0. Therefore, the relative fitness in E_0 is determined by the costs associated with switching phenotypes, so we do not observe significant effects from density dependence.

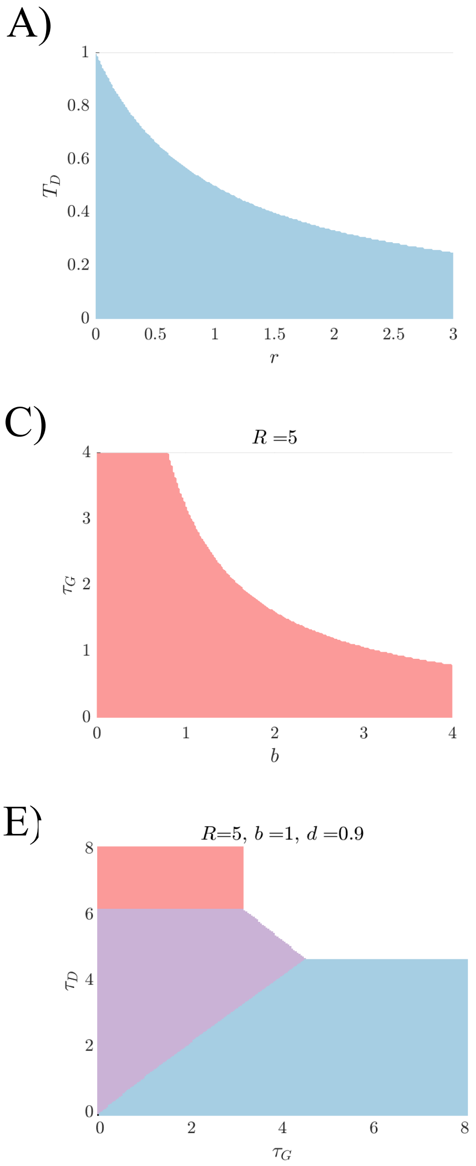

Fig. S12. Fitness comparison with density-dependence and explicit modeling of cluster sizes. The fitness comparisons in Figs 2-4 are reconstructed assuming that population growth is limited by a carrying capacity and group formation in E_A and the subsequent cell death for U and M are stochastic (modeled explicitly via an agent-based simulation). A) This shows the original fitness comparison between U and U_D from Fig 2D. B) This shows the effects on A) of incorporating density dependence and stochastic cell death. The results are qualitatively similar. C) This shows the original fitness comparison between U and M from Fig 3D. D) This shows the effects on C) of incorporating density dependence and stochastic cell death. The values for the birth rates are modified (here, we use $b > 1$) because the simulations only allow integer cell numbers, which can result in population extinction. The black color indicates simulation runs where U and M ended up with the same fitness. E) This shows the original fitness comparison between all four life cycles from Fig 4C. F) This shows the effects on E) of incorporating density dependence and stochastic cell death. The results are qualitatively similar.
